# Supplementary figures and images for: An ArfGAP-dependent signaling modulates synaptic plasticity via IP3-regulated calcium release from the endoplasmic reticulum
Source: PLoS Genet. 2026 Jan 23;22(1):e1012031. doi: 10.1371/journal.pgen.1012031 (PMC12863683; doi:10.1371/journal.pgen.1012031)

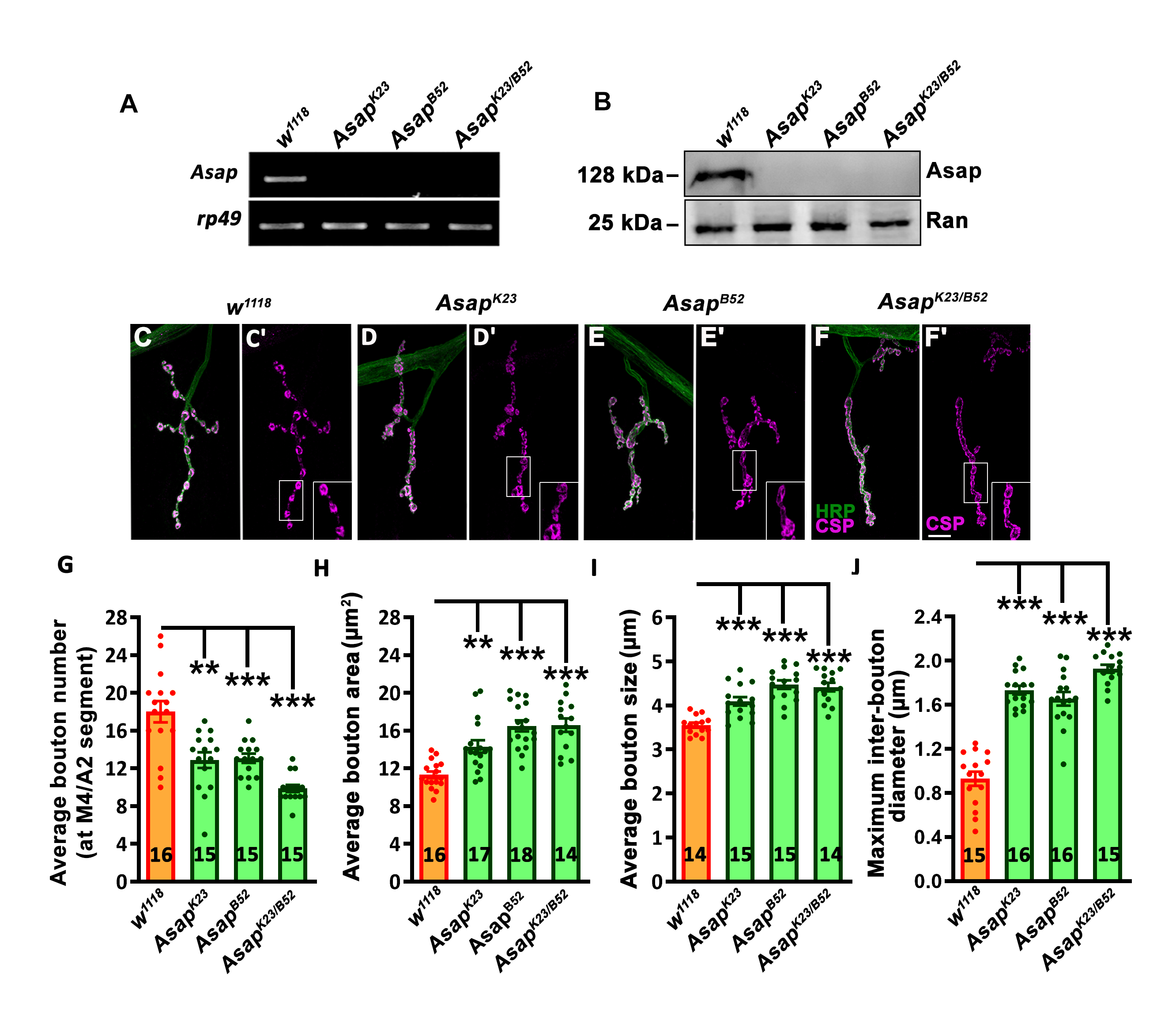

Supplement: S1 Fig — (A) Semi-quantitative RT-PCR showing Asap transcript level in w1118 controls, homozygous AsapK23, homozygous AsapB52, and heteroallelic AsapK23/B52 mutant animals. rp49 transcript levels were used as an internal loading control. (B) Western blot showing protein levels of Asap in w1118 controls, homozygous AsapK23, homozygous AsapB52, and heteroallelic AsapK23/B52 mutant animals. Ran protein levels were used as an internal loading control. (C-F′) Confocal images of NMJ synapses at muscle 4 of A2 hemisegment in (C-C′) w1118 control, (D-D′) homozygous AsapK23, (E-E′) homozygous AsapB52, and (F-F′) heteroallelic AsapK23/B52 mutant animals double immunolabeled for HRP (green) and CSP (magenta). The scale bar in F′ for (C-F′) represents 10 µm. (G-J) Histogram showing an average number of boutons (G), average bouton area (H), average bouton size (I), and maximum interbouton diameter (J) at muscle 4 NMJ of the A2 hemisegment of the indicated genotypes. **p = 0.001, ***p = 0.0001; ns, not significant. n = 14–16 NMJ per genotype. The statistical analysis was done using one-way ANOVA followed by post-hoc Tukey’s multiple-comparison test. All values represent mean ± SEM. The values for each quantification are shown in Table J in S1 Text. (TIF) [file pgen.1012031.s001.tif]

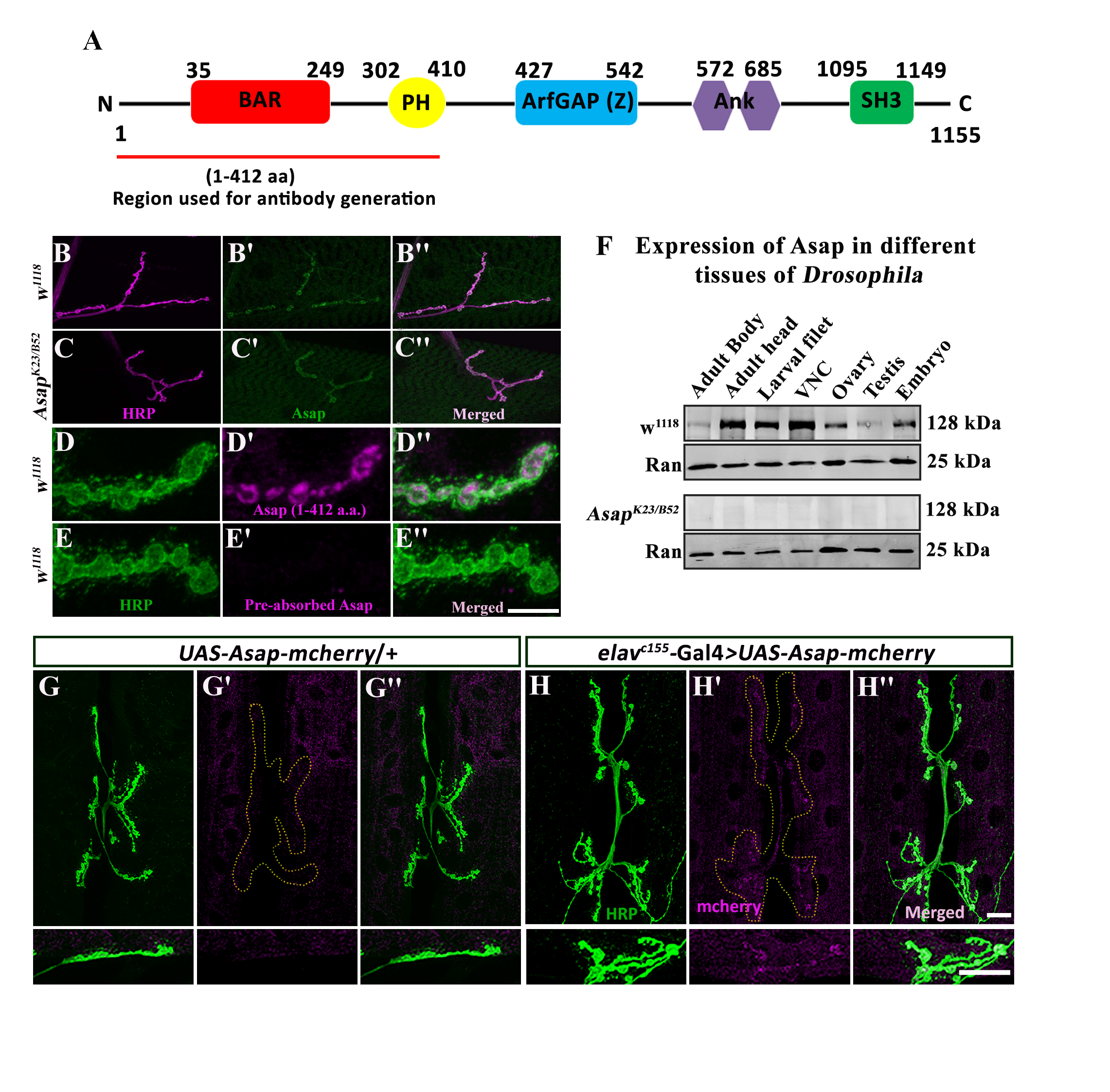

Supplement: S2 Fig — (A) Domain organization of Asap. The N-terminal 1–412 aa was used to generate antibodies against Asap. (B-C“) Confocal images of NMJ synapses at muscle 4 of A2 hemisegment in the (B-B”) wild-type (w1118) and (C-C”) AsapK23/B52 heteroallelic mutant, immunolabeled with HRP (magenta) and dAsap antibody (1–412 aa) (green). The scale bar in C” for (B-C”) is 20 µm. (D-E”) (D-D”) Represents confocal images of NMJ synapses at muscle 4 of the A2 hemisegment of wild-type, double immunostained with the Asap antibody (magenta) and HRP (green), highlighting its distribution at the NMJ. In contrast, (E-E”) represents the immunostaining of the wild-type NMJ using Asap antibody, which was preabsorbed with pure Asap protein (1–412 a.a.), resulting in no detectable staining at the NMJ. The scale bar in E” applies to panels D-E” and represents 2.5 µm. (F) Western blotting showing protein levels of Asap in different tissues in wild-type and AsapK23/B52 animals. Ran protein levels were used as an internal loading control. (G-H”) Confocal images of NMJ synapses at muscle 6–7 of the A2 hemisegment of (G-G”) UAS-Asap-mcherry/+ and (H-H”) elavc155-Gal4 > UAS-Asap-mcherry immunostained with HRP (green) and mcherry (magenta). The scale bar in H” for (G-H”) is 20 µm. (TIF) [file pgen.1012031.s002.tif]

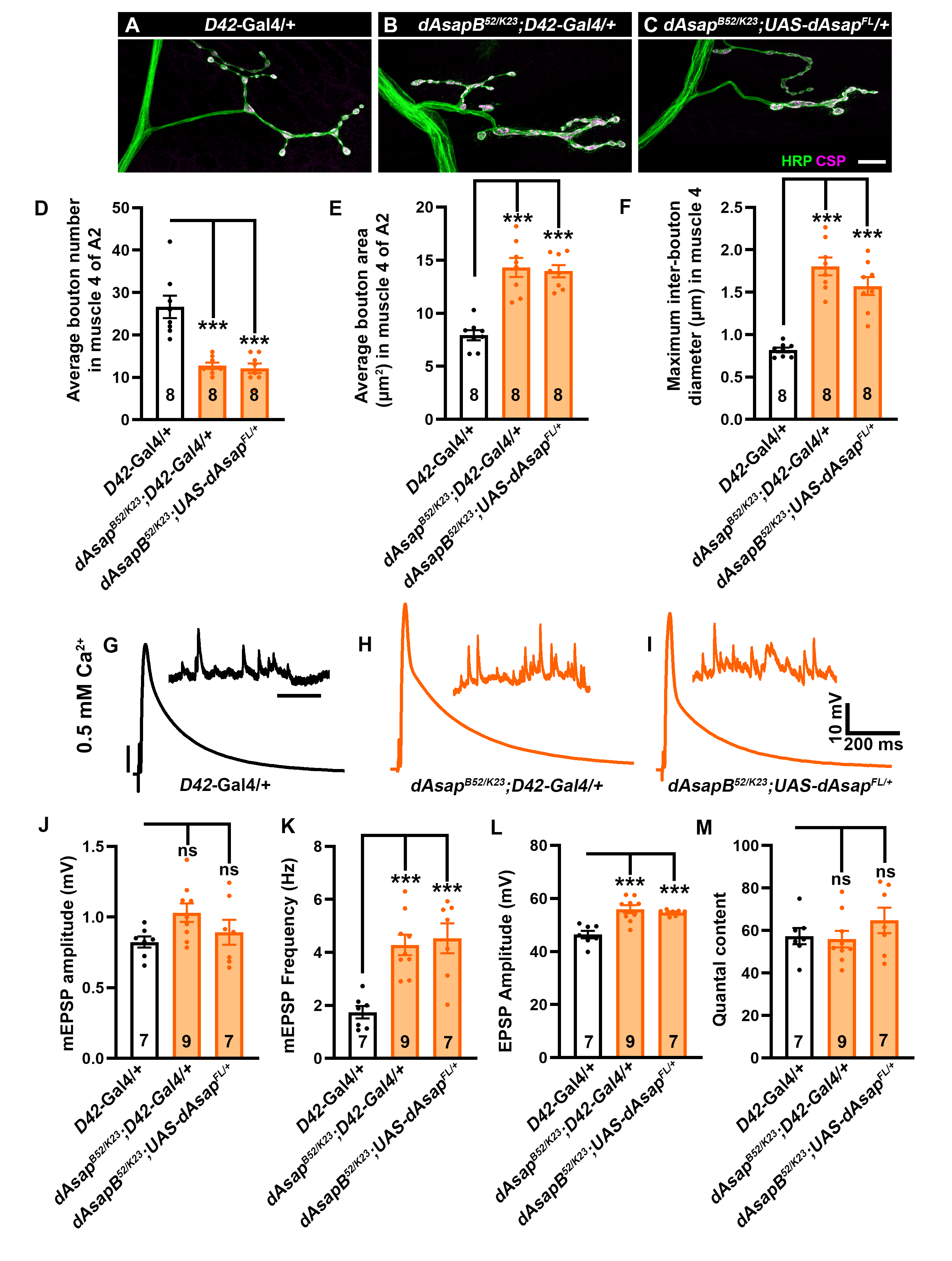

Supplement: S3 Fig — (A-C) Confocal images of NMJ synapses at muscle 4 of A2 hemisegment showing synaptic growths in (A) D42-Gal4/ + control, (B) AsapB52/K23; D42-Gal4/+ and (C) AsapB52/K23; UAS-AsapFL/ + double immunolabeled for CSP (magenta) and HRP (green). Scale bar represents 10 μm (A-C). (D-F) Histogram showing an average number of boutons (D), average bouton area (E), and maximum interbouton diameter (F) at the muscle 4 NMJ of the A2 hemisegment of the indicated genotypes. ns; not significant, ***p = 0.0002 (bouton number), ***p = 0.0001 (bouton area), ***p = 0.0001 (inter bouton diameter). The statistical analysis was done using one-way ANOVA followed by post-hoc Tukey’s multiple-comparison test. n = 8 NMJ per genotype. (G-I) Representative traces of mEPSP and EPSP in (G) D42-Gal4/ + control, (H) AsapB52/K23; D42-Gal4/+ and (I) AsapB52/K23; UAS-AsapFL/ + larvae. Scale bars for EPSPs (mEPSP) are x = 200 ms (1000 ms) and y = 10 mV (1 mV). (J-M) Histogram showing mEPSP amplitude (J), mEPSP frequency (K), EPSP amplitude (L) and Quantal content (M) from muscle 6 of A2 hemisegment in the indicated genotypes. ***p = 0.0001, ***p = 0.0006 (mEPSP frequency), ***p = 0.0005, *** p = 0.0001 (EPSP amplitude). The statistical analysis was performed using Student’s t-test for pairwise comparisons. n = 7–10 NMJ per genotype. All recordings included in the analysis have an input resistance greater than 5 MΩ. All values represent mean ± SEM. The values for each quantification are shown in Table K in S1 Text. (TIF) [file pgen.1012031.s003.tif]

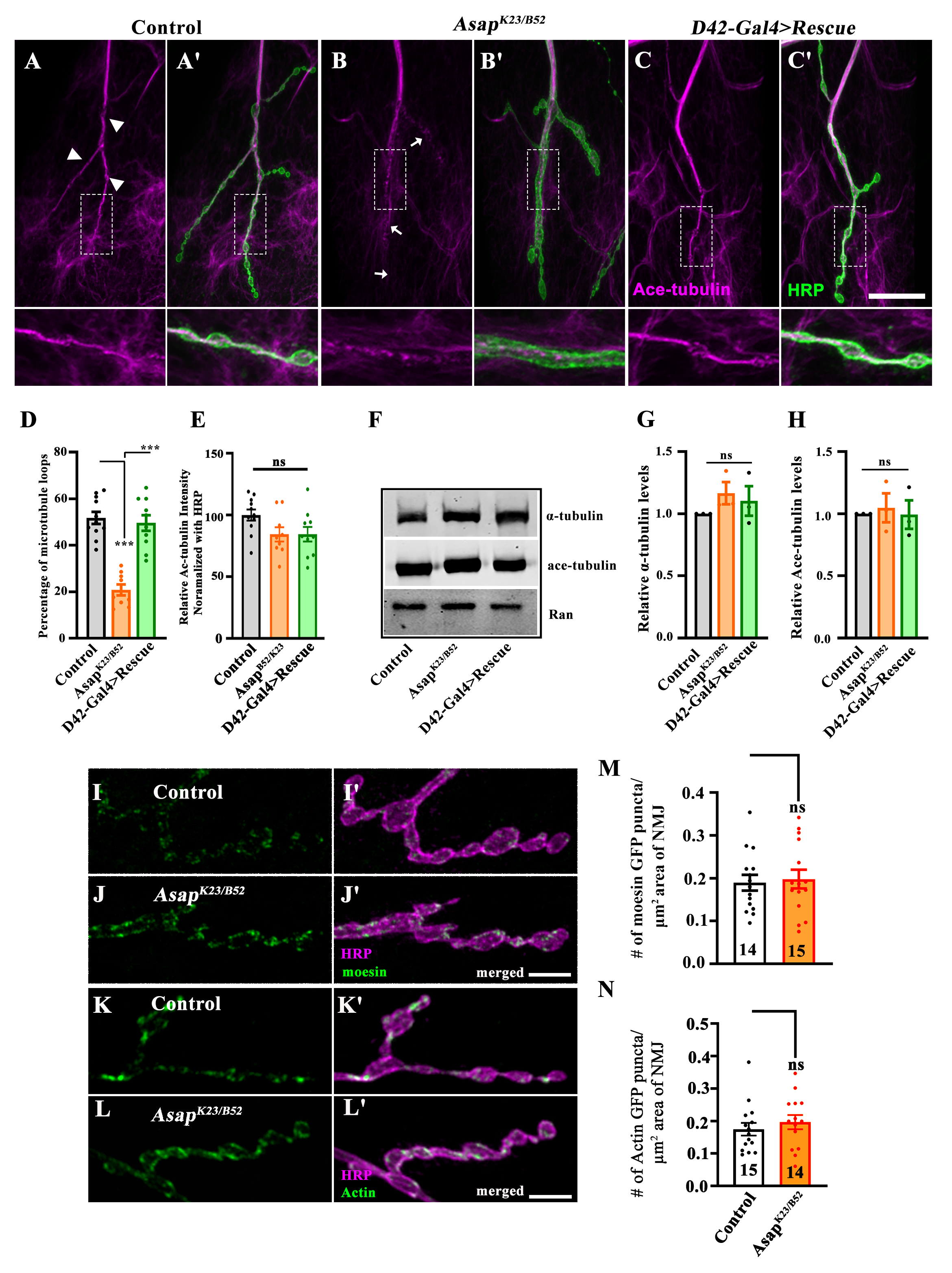

Supplement: S4 Fig — (A-C’) Representative confocal images of NMJ synapses at muscle 4 of A2 hemisegment showing the microtubule loops in (A-A’) control, (B-B’) AsapK23/B52, and (C-C’) AsapK23/B52; D42-Gal4/UAS-Asap double immunolabeled with ace-tubulin (magenta) and HRP (green). Scale bar in C′ (for A-C′) represents 10 μm. (D) Histogram showing the percentage of microtubule-positive loops from muscle 4 NMJ at A2 hemisegment in D42-Gal4/ + control (52.14 ± 2.84%), AsapK23/B52 (19.47 ± 2.23%), AsapK23/B52; D42-Gal4/UAS-Asap (48.95 ± 3.73%) animals. The error bar represents the standard error of the mean (mean ± SEM); the statistical analysis was done using one-way ANOVA followed by post-hoc Tukey’s test. ***p = 0.0001; ns, not significant. (E) Histogram showing the ace-tubulin intensity normalized with HRP from muscle 4 NMJ at A2 hemisegment in D42-Gal4/ + control, AsapK23/B52, AsapK23/B52; D42-Gal4/UAS-Asap animals. The error bar represents the standard error of the mean (SEM); the statistical analysis was done using one-way ANOVA followed by post-hoc Tukey’s test. ns, not significant. (F) Western blots showing ace-Tubulin and α-Tubulin protein levels in the indicated genotypes. Ran protein levels were used as an internal loading control. (G) Histogram showing the quantification percentage of ace-tubulin level in control, AsapK23/B52, AsapK23/B52; D42-Gal4/UAS-Asap animals. The error bar represents the standard error of the mean (SEM); the statistical analysis was done using one-way ANOVA followed by post-hoc Tukey’s test. ns, not significant. (H) Histogram showing the quantification percentage of α-tubulin level in control (1.00 ± 0.00), AsapK23/B52 (1.16 ± 0.08), AsapK23/B52; D42-Gal4/UAS-Asap (1.10 ± 0.12) animals. The error bar represents the standard error of the mean (SEM); the statistical analysis was done using one-way ANOVA followed by post-hoc Tukey’s test. ns, not significant. (I-J’) Representative confocal images of NMJ synapses at muscle 4 of A2 hemisegment showing the numb [file pgen.1012031.s004.tif]

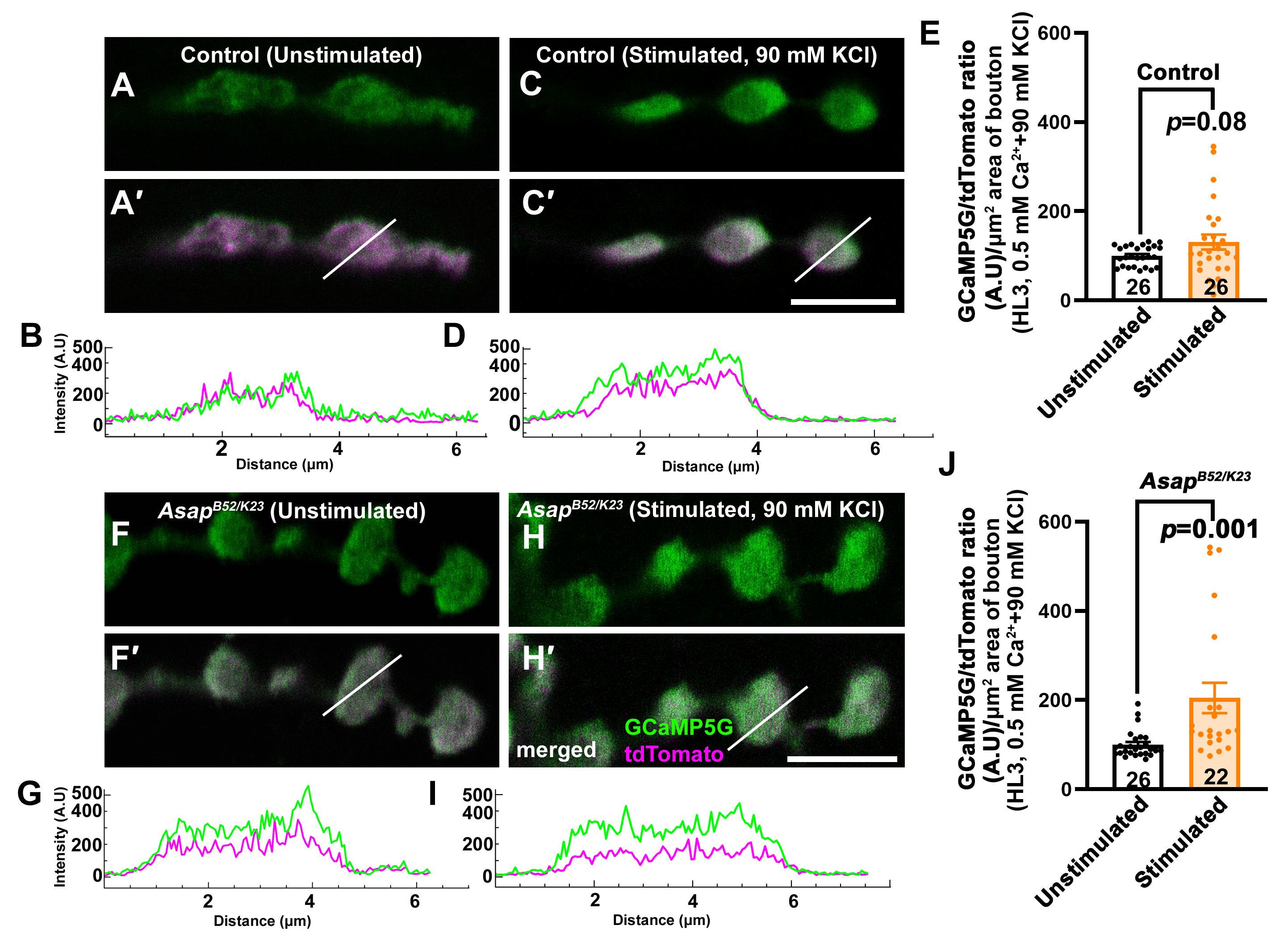

Supplement: S6 Fig — (A, A’ and C, C’) Confocal live images of NMJ synapses at muscle 6/7 of the A2 hemisegment of (A-A’) control-unstimulated and (C-C’) control-stimulated with 90 mM KCl, expressing GCaMP5G (green) and td-Tomato (magenta) fluorescent protein. The scale bar in C’ for (A- A’, C- C’) represents 4 μm. (B, D) Intensity plot profile for GCaMP5G (green) and td-Tomato (magenta) across the bouton (shown in A’ and C’ and thin line). (E) Histogram showing the fluorometric ratio of GCaMP5G and td-Tomato per µm2 bouton area in OK-371-Gal4/ + ; UAS-GCaMP5G-td-Tomato/ + control, unstimulated (100.0 ± 4.39) and OK-371-Gal4/ + ; UAS-GCaMP5G-td-Tomato/ + control, stimulated (130.79 ± 16.73) larvae. p = 0.08. The statistical analysis was performed using Student’s t-test for pairwise comparisons. n = 26 boutons per genotype. All values represent mean ± SEM. (F, F’ and H-H’) Confocal live images of NMJ synapses at muscle 6/7 of A2 hemisegment of (F-F’) AsapB52/K23-unstimulated and (H-H’) AsapB52/K23-stimulated with 90 mM KCl expressing GCaMP5G (green) and td-Tomato (magenta) fluorescent protein. The scale bar in H’ for (F- F’, H- H’,) represents 4 μm. (G, I) Intensity plot profile for GCaMP5G (green) and td-Tomato (magenta) across the bouton (shown in F’ and H’ and thin line). (J) Histogram showing the fluorometric ratio of GCaMP5G and td-Tomato per µm2 bouton area in OK371-Gal4, AsapB52/K23; UAS-GCaMP5G-td-Tomato/ + , unstimulated (100.0 ± 6.00) and OK371-Gal4, AsapB52/K23; UAS-GCaMP5G-td-Tomato/ + , stimulated (204.7 ± 33.81) larvae. p = 0.001. The statistical analysis was performed using Student’s t-test for pairwise comparisons. n = 22–26 boutons per genotype. All values represent mean ± SEM. The values for each quantification are shown in Table N in S1 Text. (TIF) [file pgen.1012031.s006.tif]

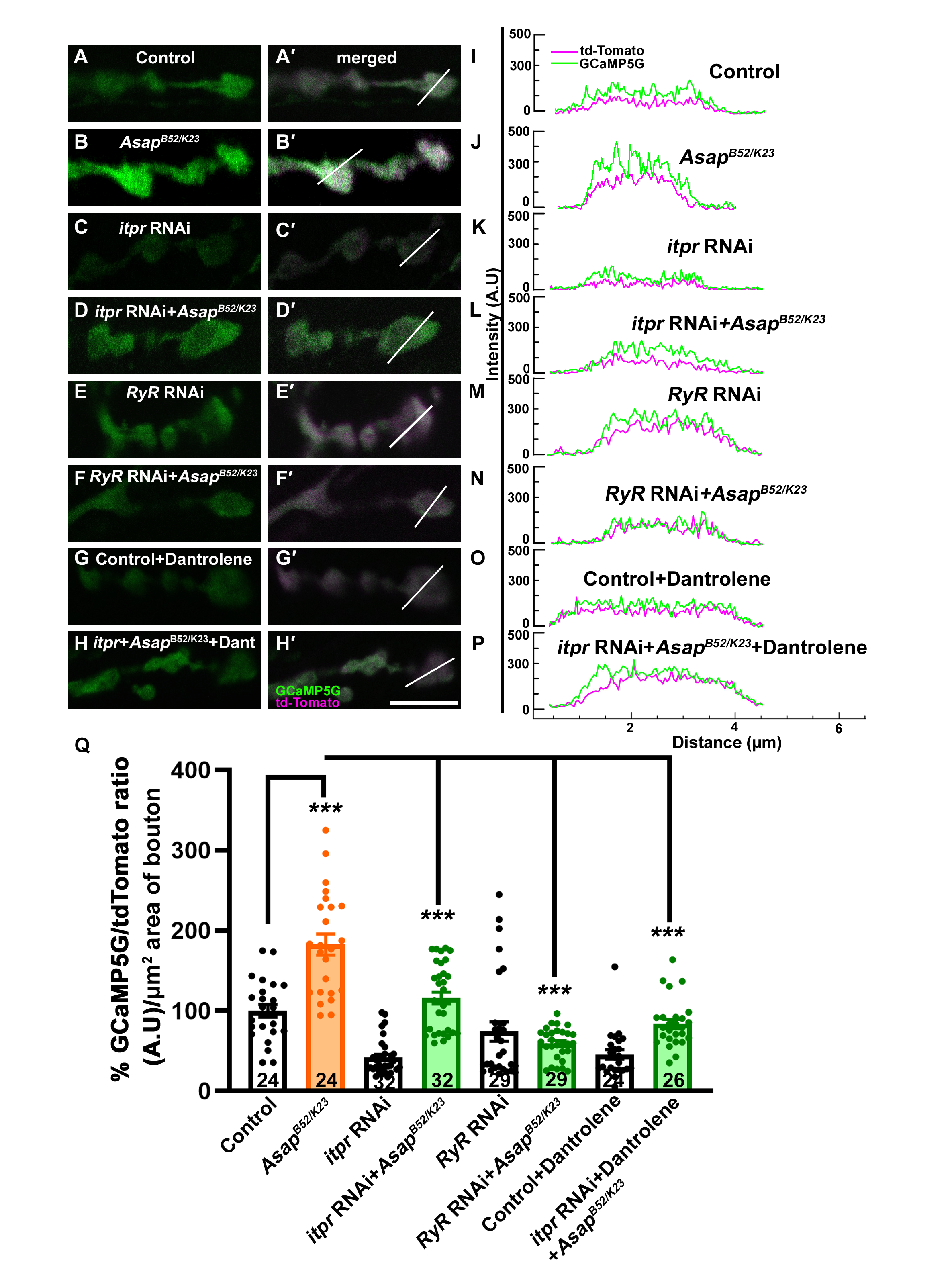

Supplement: S7 Fig — (A-H’) Confocal live images of NMJ synapses at muscle 6/7 of A2 hemisegment of (A-A’) OK-371-Gal4/ + ; UAS-td-Tomato-GCaMP5G/ + , (B-B′) OK371-Gal4, AsapB52/K23; UAS-td-Tomato-GCaMP5G/ + , (C-C′) OK371-Gal4/ + ; UAS-td-Tomato-GCaMP5G/UAS-itpr RNAi, (D-D′) OK371-Gal4, AsapB52/OK371-Gal4, AsapK23; UAS-td-Tomato-GCaMP5G/UAS-itpr RNAi, (E-E′) OK371-Gal4/ + ; UAS-td-Tomato-GCaMP5G/ UAS-RyR RNAi, (F-F′) OK371-Gal4, AsapB52/OK371-Gal4, AsapK23; UAS-td-Tomato-GCaMP5G/UAS-RyR RNAi, (G-G′) OK-371-Gal4/ + ; UAS-td-Tomato-GCaMP5G/ + Dantrolene and (H-H′) OK371-Gal4, AsapB52/OK371-Gal4, AsapK23; UAS-td-Tomato-GCaMP5G/UAS-itpr RNAi + Dantrolene expressing GCaMP5G (green) and td-Tomato (magenta) fluorescent protein. (I-P) Intensity plot profile for GCaMP5G (green) and tdTomato (magenta) across the bouton (shown in A’-H’) as a thin line. The scale bar in H’ for (A-H’) represents 4 μm. (Q) Histogram showing the % of fluorescence ratio of GCaMP5G and td-Tomato per µm2 bouton area in the indicated genotypes. ***p = 0.0001. The statistical analysis was done using one-way ANOVA followed by post-hoc Tukey’s multiple-comparison test. n = 24–32 boutons per genotype. All values represent mean ± SEM. The values for each quantification are shown in Table O in S1 Text. (TIF) [file pgen.1012031.s007.tif]

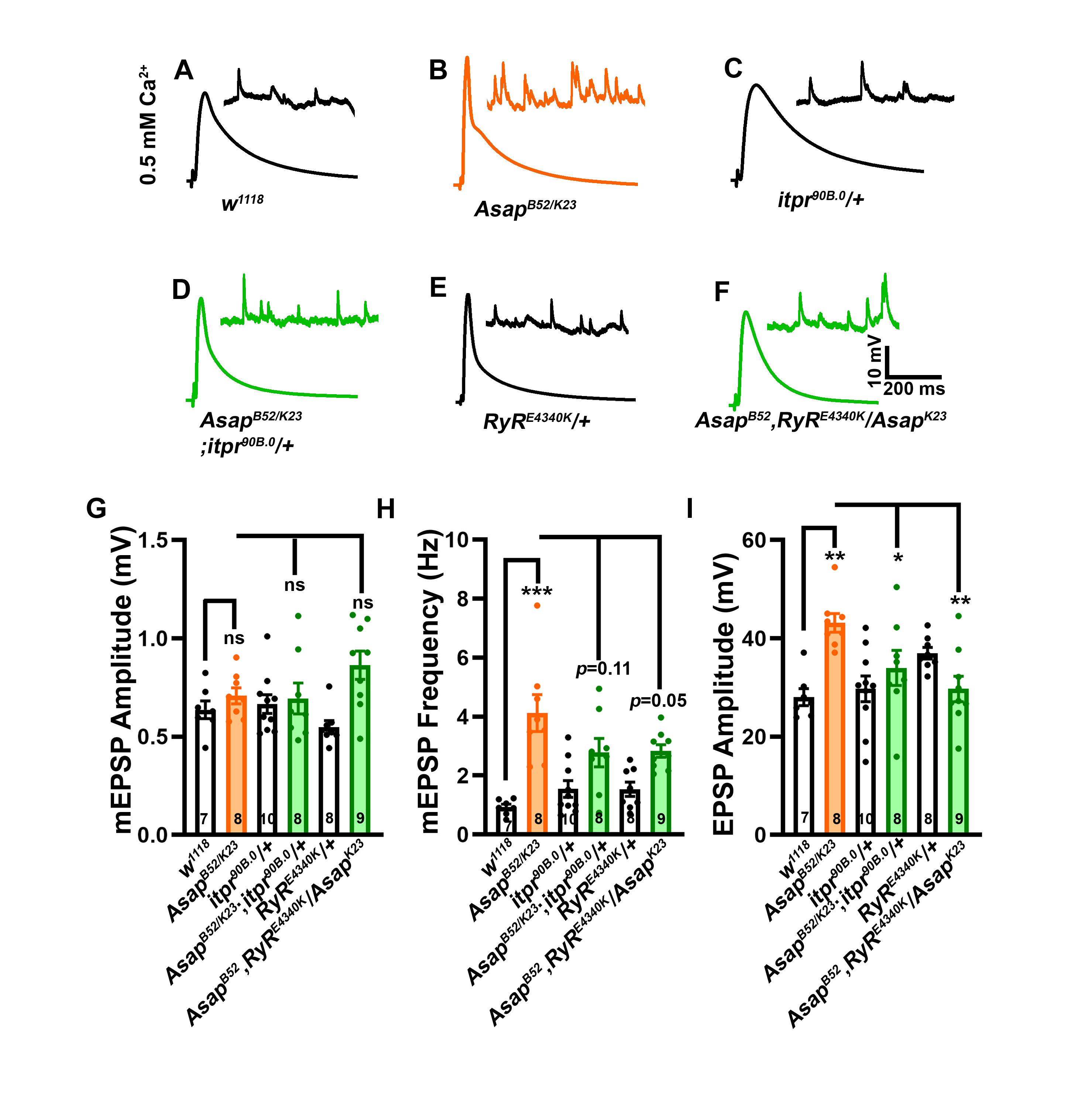

Supplement: S8 Fig — (A-F) Representative traces of mEPSP and EPSP in (A) w1118 control, (B) AsapB52/K23, (C) itpr90B.0/ + , (D) AsapB52/K23; itpr 90B.0/ + , (E) RyRE4340K/+ and (F) AsapB52, RyRE4340K/AsapK23 larvae. Scale bars for EPSPs (mEPSP) are x = 200 ms (1000 ms) and y = 10 mV (1 mV). (G-I) Histogram showing mEPSP amplitude (G), mEPSP frequency (H) and EPSP amplitude (I) from muscle 6 of A2 hemisegment in the indicated genotypes. ***p = 0.0004 (mEPSP frequency), *p = 0.03, **p = 0.001 (EPSP amplitudes). The error bar represents the standard error of the mean (SEM). The statistical analysis was done using one-way ANOVA followed by post-hoc Tukey’s test. ns, not significant. n = 7–10 NMJ per genotype. All recordings included in the analysis have an input resistance greater than 5 MΩ. The values for each quantification are shown in Table P in S1 Text. (TIF) [file pgen.1012031.s008.tif]

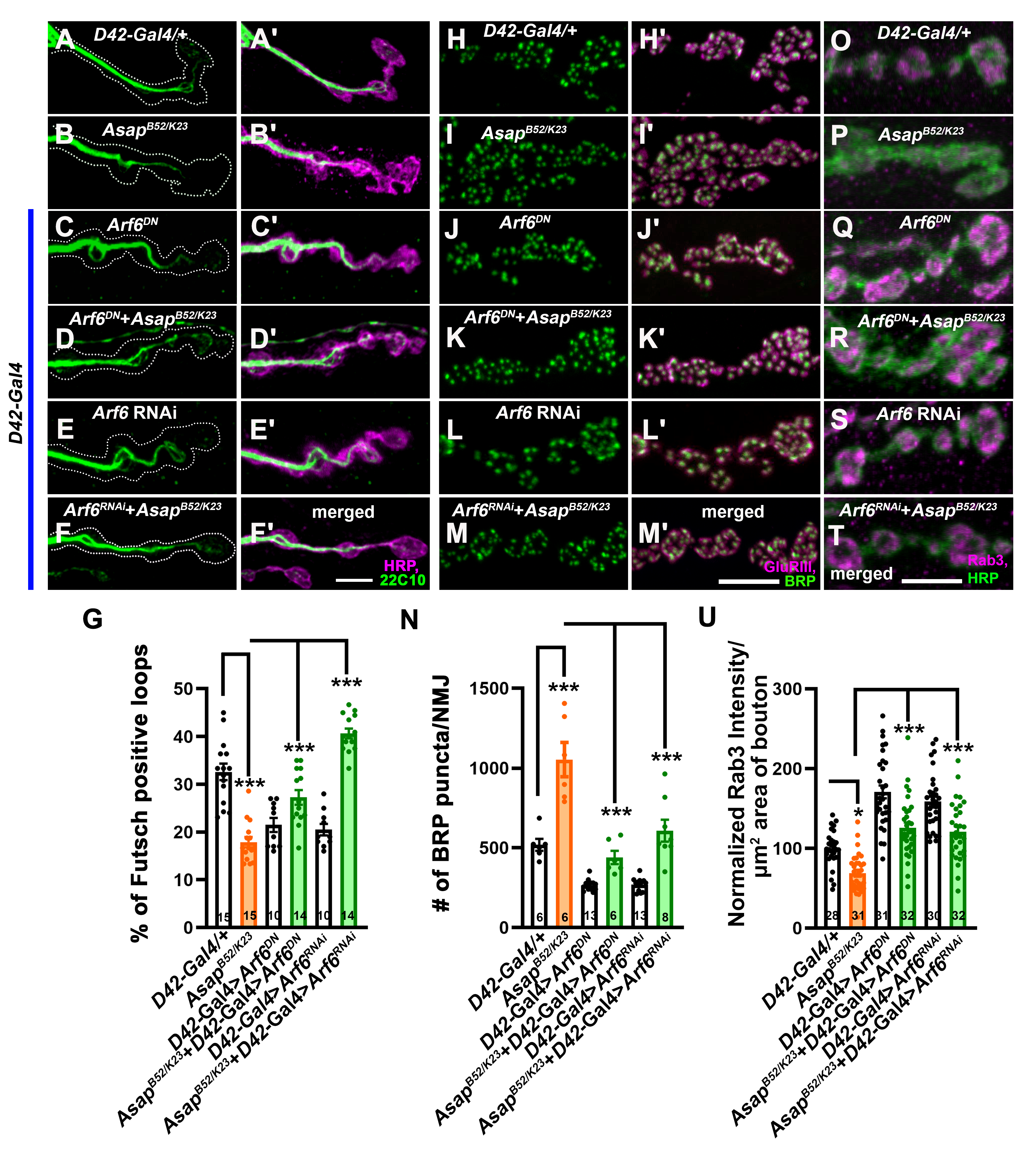

Supplement: S9 Fig — (A-F’) Representative confocal images of NMJ synapses at muscle 4 of A2 hemisegment showing the futsch loops in (A-A’) D42-Gal4/ + control, (B-B’) AsapK23/B52, (C-C’) D42-Gal4/UAS-Arf6DN, (D-D’) AsapK23/B52; D42-Gal4/UAS-Arf6DN, (E-E’) UAS-Arf6 RNAi/ + ; D42-Gal4/+ and (F-F’) UAS-Arf6 RNAi, AsapB52/K23; D42-Gal4/ + double immunolabeled with 22C10 (green) and a neuronal membrane marker, HRP (magenta). Scale bar in F′ (for A-F′) represents 4 μm. (G) Histogram showing the percentage of futsch positive loops from muscle 4 NMJ at A2 hemisegment in D42-Gal4/ + control, AsapK23/B52, D42-Gal4/UAS-Arf6DN, AsapK23/B52; D42-Gal4/UAS-Arf6DN, UAS-Arf6RNAi/ + ; D42-Gal4/+ and UAS-Arf6 RNAi, AsapB52/K23; D42-Gal4/ + animals. The error bar represents the standard error of the mean (SEM); the statistical analysis was done using one-way ANOVA followed by post-hoc Tukey’s test. ***p = 0.0001; ns, not significant. n = 10–15 NMJ per genotype. All values represent mean ± SEM. (H-M’) Representative confocal images of NMJ synapses at muscle 4 of A2 hemisegment showing active zone density in (H-H’) D42-Gal4/ + control, (I-I’) AsapK23/B52, (J-J’) D42-Gal4/UAS-Arf6DN, (K-K’) AsapK23/B52; D42-Gal4/UAS-Arf6DN, (L-L’) UAS-Arf6RNAi/ + ; D42-Gal4/+ and (M-M’) UAS-Arf6 RNAi, AsapB52/K23; D42-Gal4/ + double immunolabeled with antibodies against active zone marker Brp (green) and GluRIII (magenta). The scale bar in M′ (for H-M′) represents 2.5 μm. (N) Histogram showing the number of Brp punctae per NMJ from muscle 4 NMJ at A2 hemisegment in D42-Gal4/ + control, AsapK23/B52, D42-Gal4/UAS-Arf6DN, AsapK23/B52; D42-Gal4/UAS-Arf6DN, UAS-Arf6RNAi/ + ; D42-Gal4/+ and UAS-Arf6 RNAi, AsapB52/K23; D42-Gal4/ + animals. The error bar represents the standard error of the mean (SEM); the statistical analysis was done using one-way ANOVA followed by post-hoc Tukey’s test. ***p < 0.0001; ns, not significant. n = 6–13 NMJ per genotype. All values represent mean ± SEM. (O-T) Representative confocal images of NMJ synap [file pgen.1012031.s009.tif]

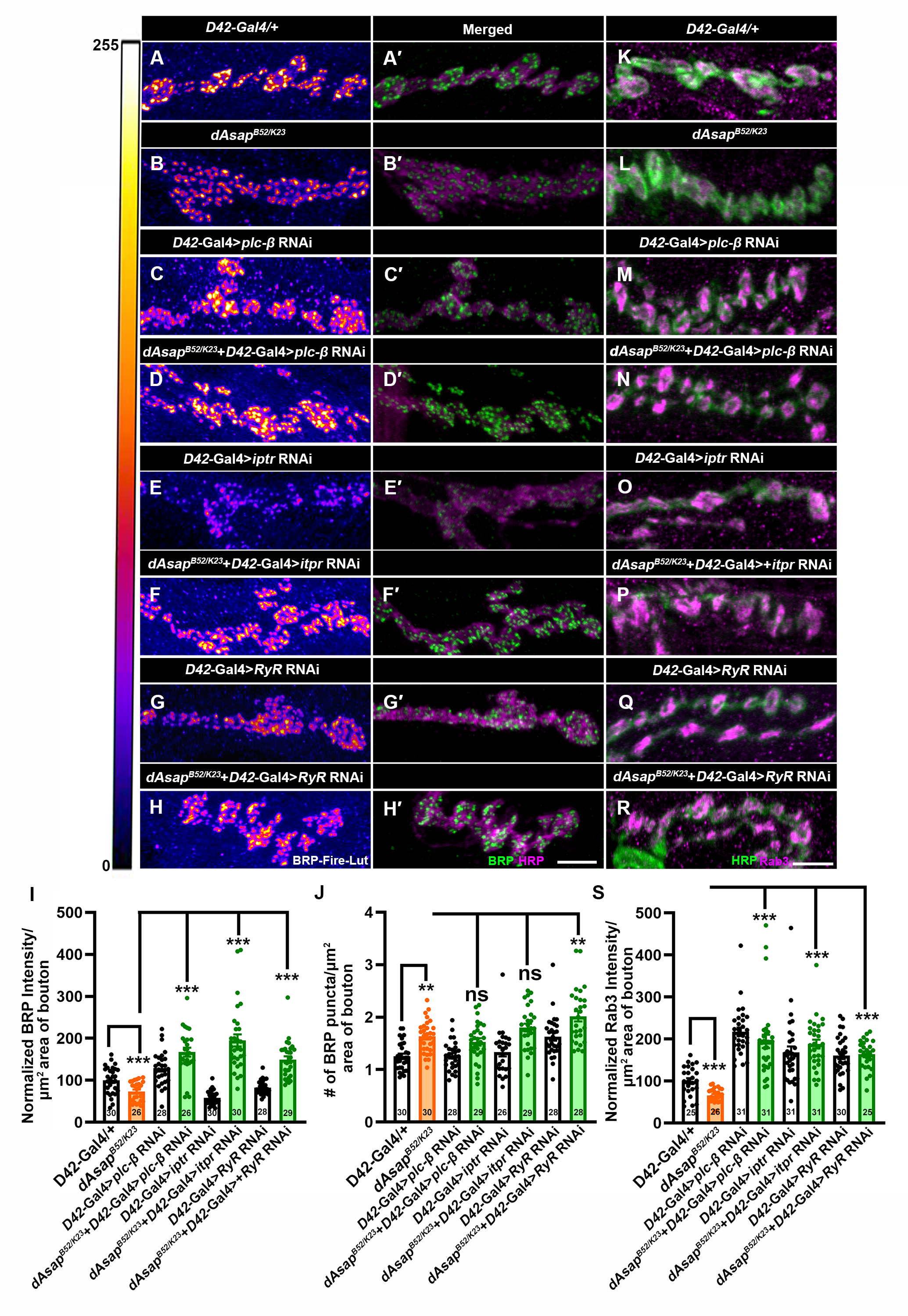

Supplement: S10 Fig — (A-H’) Confocal images of NMJ synapses at muscle 4 of A2 hemisegment in D42-Gal4/+ (A-A’), AsapB52/K23 (B-B’), D42-Gal4/UAS-plc-β RNAi (C-C’), AsapB52/K23; D42-Gal4/UAS-plc-β RNAi (D-D’), D42-Gal4/UAS-itpr RNAi (E-E’), AsapB52/K23; D42-Gal4/UAS-itpr RNAi (F-F’), D42-Gal4/UAS-RyR RNAi (G-G’) and AsapB52/K23; D42-Gal4/UAS-RyR RNAi (H-H’) double immunolabeled with antibodies against active zones marker Bruchpilot, Brp (green) and HRP (magenta). The scale bar in H’ represents 2.5 μm for A-H’. (I) Histogram showing the levels of Brp per μm2 area of bouton from muscle 4 at the A2 hemisegment in the indicated genotypes. ***p = 0.001, ***p = 0.0001. n = 26–30 boutons per genotype. All values represent mean ± SEM. (J) Histogram showing the number of Brp punctae per μm2 area of bouton from muscle 4 NMJ at A2 hemisegment in the indicated genotypes. **p = 0.01, n = 26–30 boutons per genotype. All values represent mean ± SEM. (K-R) Confocal images of NMJ synapses at muscle 4 of A2 hemisegment in D42-Gal4/+ (K), AsapB52/K23 (L), D42-Gal4/UAS-plc-β RNAi (M), AsapB52/K23; D42-Gal4/UAS-plc-β RNAi (N), D42-Gal4/UAS-itpr RNAi (O), AsapB52/K23; D42-Gal4/UAS-itpr RNAi (P), D42-Gal4/UAS-RyR RNAi (Q) and AsapB52/K23; D42-Gal4/UAS-RyR RNAi (R) double immunolabeled with antibodies against Rab3 (magenta) and HRP (green). The scale bar in R represents 2.5 μm for K-R. (S) Histogram showing the levels of Rab3 per μm2 area of bouton from muscle 4 NMJ at A2 hemisegment in the indicated genotypes. ***p = 0.001. n = 25–31 boutons per genotype. All values represent mean ± SEM. The values for each quantification are shown in Table R in S1 Text. (TIF) [file pgen.1012031.s010.tif]

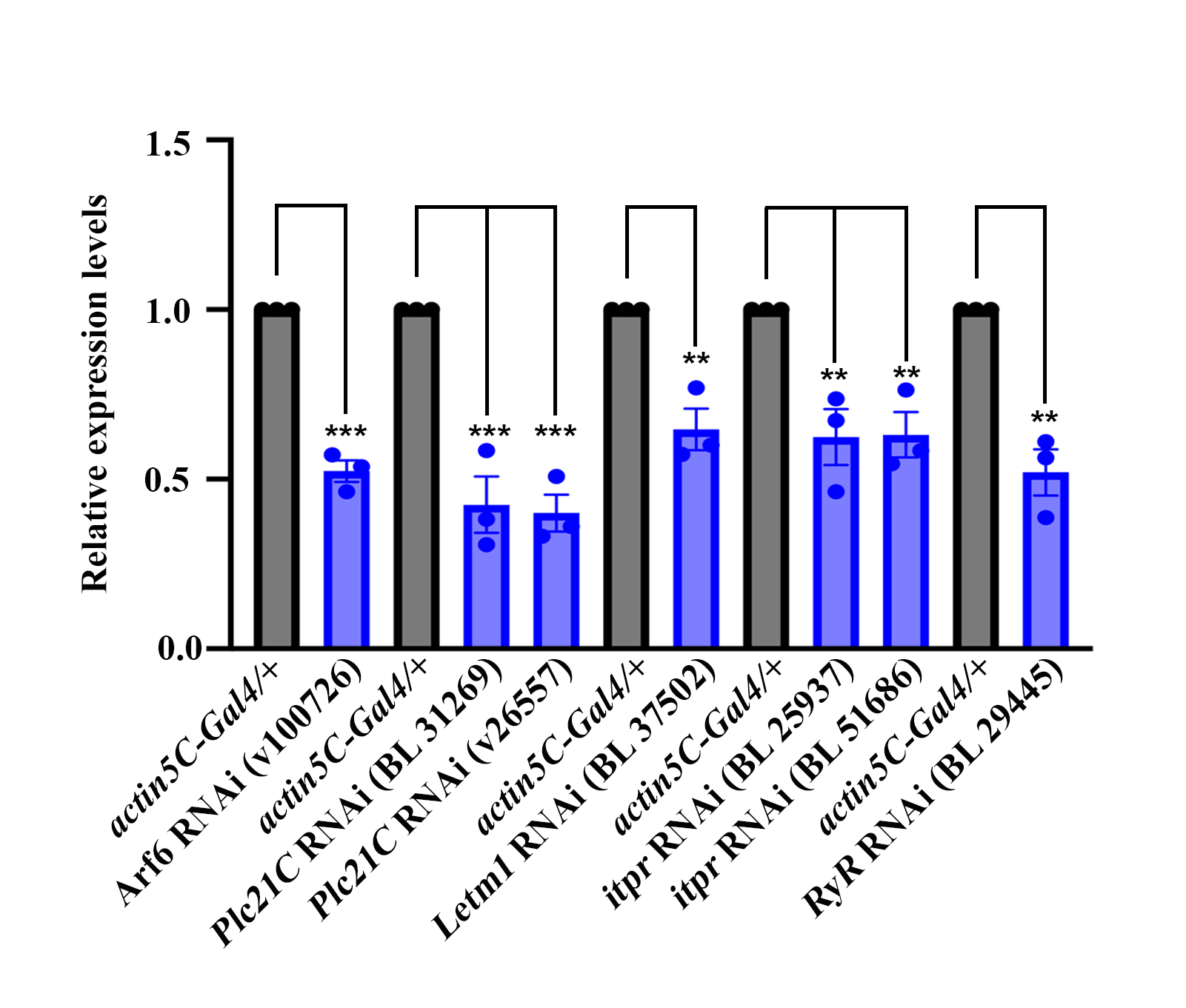

Supplement: S11 Fig — Quantitative RT-PCR showing transcript levels of Arf6, Plc21C, Letm1, Itpr, and RyR in actin5C-Gal4-driven corresponding RNAi lines, as indicated in the figure. The RNAi lines for Arf6, Plc21C, and RyR showed a ~ 50% reduction in transcript levels compared to the control animals. For Itpr and Letm1, a ~ 40% reduction in transcript levels was observed compared to the actin5C-Gal4 control (actin5C-Gal4/+). All values represent mean ± SEM. **p = 0.01, ***p = 0.001. The values for each quantification are shown in Table S in S1 Text. (TIF) [file pgen.1012031.s011.tif]
