# Supplementary material for: An ArfGAP-dependent signaling modulates synaptic plasticity via IP3-regulated calcium release from the endoplasmic reticulum
Source: PLoS Genet. 2026 Jan 23;22(1):e1012031. doi: 10.1371/journal.pgen.1012031 (PMC12863683; doi:10.1371/journal.pgen.1012031)
Supplement: S1 Text — The table shows the analysis of total bouton number, average bouton area, average bouton size, average inter-bouton diameter, and Futsch loops on muscle 4 of A2 hemisegment in various genetic combinations as indicated. Values are represented as mean±s.e.m. The control mean is compared with the mutant, while the mutant mean is compared with various rescue combinations. These values relate to Fig 1. Table B. The table shows values of mEPSP amplitude, mEPSP frequency, EPSP amplitude, Quantal content, the average number of Brp punctae per NMJ, GluRIII cluster area, number of synaptic failure events, Brp intensity/ µm2 area of bouton, CAC intensity/ µm2 area of bouton, and CAC density µm2 area of bouton in the indicated genotypes. Values are represented as mean±s.e.m. The control mean is compared with the mutant, while the mutant mean is compared with the rescue combination. These values relate to Fig 2. (DOCX) [file pgen.1012031.s012.docx]

**An ArfGAP-Dependent Signaling Modulates Synaptic Plasticity via IP3-Regulated Calcium Release from the Endoplasmic Reticulum**

This is the S1 Text for Mallik *et al.* It contains the following:

- Tables A-S: Summary data for graphs in Main Figures and Supplemental Figures.
- Table T: Detailed information about the primers used for the RT-qPCR.

| **Sl**  **No.** | **Genotypes** | **# Boutons** | **Bouton area**  **(in µm^2^)** | **Bouton size**  **(in µm)** | **Inter-bouton**  **diameter**  **(in µm)** | **% of Futsch**  **positive loops** |
| --- | --- | --- | --- | --- | --- | --- |
| **1** | ***D42-Gal4/+*** | 18.18 ± 0.75 | 9.30 ± 0.42 | 3.55 ± 0.05 | 0.88 ± 0.03 | 35.48 ± 2.40 |
| **2** | ***Asap^B52/K23^*** | 11.63 ± 0.42 | 12.79 ± 0.49 | 4.41 ± 0.09 | 1.83 ± 0.05 | 23.61 ± 2.40 |
| **3** | ***actin5C-Gal4,***  ***Asap^K23/B52^;***  ***UAS-Asap^FL^/+*** | 17.68 ± 0.81 | 10.65 ± 0.52 | 3.55 ± 0.08 | 1.08 ± 0.05 | 33.57 ± 2.15 |
| **4** | ***Asap^B52/K23^; UAS-***  ***Asap^FL^/D42-Gal4*** | 17.93 ± 0.81 | 9.02 ± 0.40 | 3.77 ± 0.06 | 1.10 ± 0.03 | 39.20 ± 2.68 |
| **5** | ***Asap^B52/K23^;***  ***UAS-Asap^FL^***  ***/mef2-Gal4*** | 12.17 ± 0.45 | 13.66 ± 0.61 | 4.42 ± 0.10 | 1.71 ± 0.05 | 23.14 ± 2.28 |

**Table A.** The table shows the analysis of total bouton number, average bouton area, average bouton size, average inter-bouton diameter, and Futsch loops on muscle 4 of A2 hemisegment in various genetic combinations as indicated. Values are represented as mean±s.e.m. The control mean is compared with the mutant, while the mutant mean is compared with various rescue combinations. These values relate to **Fig 1**.

| **Sl**  **No.** | **Genotypes** | **mEPSP**  **amplitude**  **(mV)** | **mEPSP**  **frequency**  **(Hz)** | **EPSP**  **amplitude**  **(mV)** | **Quantal**  **content**  **(QC)** |
| --- | --- | --- | --- | --- | --- |
| **1** | ***D42-Gal4/+*** | 0.60 ± 0.04 | 1.61 ± 0.17 | 51.45 ± 1.27 | 88.60 ± 5.27 |
| **2** | ***Asap^K23/B52^*** | 0.73 ± 0.04 | 4.82 ± 0.39 | 60.10 ± 2.48 | 83.63 ± 3.97 |
| **3** | ***Asap^B52/K23^;***  ***D42-Gal4/UAS-Asap^FL^*** | 0.68 ± 0.04 | 2.77 ± 0.31 | 52.47 ± 1.82 | 80.27 ± 4.36 |
| **Sl**  **No.** | **Genotypes** | **# of BRP puncta/NMJ** | **# of BRP puncta/ µm^2^ area of bouton** | **GluRIII**  **cluster area**  **(µm^2^)** | **# of failures/100 events** |
| **1** | ***D42-Gal4/+*** | 404.2 ±19.09 | 1.79 ± 0.05 | 0.48 ± 0.03 | 28.55 ± 9.04 |
| **2** | ***Asap^K23/B52^*** | 625.9± 39.98 | 2.20 ± 0.06 | 1.29 ± 0.06 | 7.53 ± 3.17 |
| **3** | ***Asap^B52/K23^;***  ***D42-Gal4/UAS-Asap^FL^*** | 476.6 ± 14.90 | 1.93 ± 0.06 | 0.60 ± 0.04 | 23.10 ± 6.71 |
| **Sl**  **No.** | **Genotypes** | **BRP intensity (A.U)/µm^2^ area of bouton** | **Genotypes** | **CAC intensity (A.U)/µm^2^ area of bouton** | **# of CAC puncta/µm^2^ area of bouton** |
| **1** | ***D42-Gal4/+*** | 100.0 ± 4.20 | **1. *Cac sfGFP-N/+; OK-371-Gal4/+*** | 100.0 ± 7.21 | 0.74 ± 0.03 |
| **2** | ***Asap^K23/B52^*** | 56.67 ± 4.53 | **2. *Cac sfGFP-N/+; OK371-Gal4, Asap^K23/B52^*** | 49.87 ± 3.26 | 1.04 ± 0.03 |
| **3** | ***Asap^B52/K23^;***  ***D42-Gal4/UAS-Asap^FL^*** | 83.39 ± 2.92 | **3. *Cac sfGFP-N/+; OK371-Gal4, Asap^K23/B52^; UAS-Asap^FL^/+*** | 170.02 ± 7.21 | 0.81 ± 0.04 |

**Table B.** The table shows values of mEPSP amplitude, mEPSP frequency, EPSP amplitude, Quantal content, the average number of Brp punctae per NMJ, GluRIII cluster area, number of synaptic failure events, Brp intensity/ µm^2^ area of bouton, CAC intensity/ µm^2^ area of bouton, and CAC density µm^2^ area of bouton in the indicated genotypes. Values are represented as mean±s.e.m. The control mean is compared with the mutant, while the mutant mean is compared with the rescue combination. These values relate to **Fig 2**.

| **Sl**  **No.** | **Genotypes** | **mEPSP**  **amplitude**  **(mV)** | **mEPSP**  **frequency**  **(Hz)** | **EPSP amplitude**  **(mV)** | **Quantal content**  **(QC)** | **BRP Intensity/**  **μm2 area of bouton** |
| --- | --- | --- | --- | --- | --- | --- |
| **1** | **Control** | 0.73 ± 0.07 | 2.67 ± 0.22 | 40.28 ± 1.45 | 100.0 ± 10.07 | 100.0 ± 5.21 |
| **2** | **Control+PhTx** | 0.45 ± 0.04 | 1.96 ± 0.16 | 40.62 ± 1.46 | 163.2 ± 15.46 | 162.2 ± 9.91 |
| **3** | ***Asap^K23/B52^*** | 0.79 ± 0.05 | 4.06 ± 0.35 | 49.21 ± 1.87 | 100.0 ± 5.17 | 100.0 ± 8.48 |
| **4** | ***Asap^K23/B52^*+PhTx** | 0.57 ± 0.02 | 1.64 ± 0.31 | 48.12 ± 1.03 | 133.9 ± 6.50 | 156.7 ± 11.47 |

**Table C.** The table shows values of mEPSP amplitude, mEPSP frequency, EPSP amplitude, Quantal content, and Brp intensity in the indicated genotypes. Values are represented as mean±s.e.m. The control mean is compared with Control+PhTx, while the *Asap^K23/B52^* mean is compared with the *Asap^K23/B52^*+PhTx mean values. These values relate to **Fig 3**.

| **Sl**  **No.** | **Genotypes** | **% GCaMP5G/td-Tomato ratio (A.U)/µm^2^ area of bouton** | |
| --- | --- | --- | --- |
| **1** | ***OK-371-Gal4/+; UAS-GCaMP5G-td-Tomato/+*** | 100.0 ± 8.57 | |
| **2** | ***OK-371-Gal4, Asap^K23/B52^; UAS-GCaMP5G-td-Tomato/+*** | 194.3 ± 25.96 | |
| **Sl**  **No.** | **Genotypes** | **mEPSP freq.**  **(Hz)** | **EPSP amplitude**  **(mV)** |
| **1** | **Control+DMSO+wash** | 2.20 ± 0.39 | 39.54 ± 1.34 |
| **2** | ***Asap^B52/K23^+*DMSO*+***  **wash** | 4.61 ± 0.51 | 43.99 ± 0.90 |
| **3** | **Control+BAPTA-AM+**  **wash** | 2.16 ± 0.19 | 38.51 ± 1.48 |
| **4** | ***Asap^B52/K23^+***  **BAPTA-AM*+* wash** | 1.47 ± 0.07 | 27.13 ± 3.58 |
| **Sl**  **No.** | **Genotypes** | **mEPSP**  **frequency**  **(Hz)** | **EPSP amplitude**  **(mV)** |
| **1** | ***D42-Gal4/+* (Control)** | 1.61 ± 0.16 | 31.48 ± 1.95 |
| **2** | ***UAS-IP_3_-sponge.m30/***  ***D42-Gal4*** | 1.40 ± 0.13 | 41.52 ± 1.66 |
| **3** | ***RyR RNAi/D42-Gal4*** | 1.09 ± 0.06 | 43.93 ± 2.03 |
| **4** | ***Letm1 RNAi/+; D42-Gal4/+*** | 2.25 ± 0.20 | 42.39 ± 1.80 |
| **5** | ***Asap^B52/K23^*** | 3.08 ± 0.22 | 45.16 ± 1.11 |
| **6** | ***Asap^B52/K23^*; *UAS-IP_3_-sponge.m30/D42-Gal4*** | 1.77 ± 0.19 | 36.66 ± 1.31 |
| **7** | ***Asap^B52/K23^*; *RyR RNAi***  ***/D42-Gal4*** | 1.76 ± 0.18 | 40.11 ± 1.26 |
| **8** | ***Asap^B52/K23^;***  ***UAS-IP_3_-sponge.m30,***  ***D42-Gal4***  ***/RyR RNAi, D42-Gal4*** | 1.79 ± 0.23 | 26.75 ± 1.63 |
| **9** | ***Asap^B52/K23^; Letm1 RNAi/***  ***D42-Gal4*** | 3.88 ± 0.46 | 45.62 ± 1.47 |

**Table D.** The table shows values of % of GCaMP5G/td-Tomato ratio (A.U.)/µm^2^ area of bouton in various genetic combinations as indicated. The table shows values of mEPSP frequency and EPSP amplitude of the above genotypes. Values are represented as mean±s.e.m. For pharmacological (BAPTA-AM) experiments, the Control+DMSO+wash mean is compared with *Asap^B52/K23^+*DMSO*+* wash*,* while the Control+BAPTA-AM+wash mean is compared with *Asap^B52/K23^+*BAPTA-AM*+* wash. Similarly, for genetic experiments, the Control mean is compared with *the UAS-IP_3_-sponge.m30/D42-Gal*, *RyR RNAi/D42-Gal4*, *Letm1 RNAi/D42-Gal4* and *Asap ^B52/K23^* mean values, while *Asap ^B52/K23^* mean is compared with *Asap ^B52/K23^*; *UAS-IP_3_-sponge.m30/D42-Gal4*, *Asap ^B52/K23^*; *RyR RNAi/D42-Gal4*, *Asap^B52/K23^;UAS-IP_3_-sponge.m30,D42-Gal4/RyR RNAi,D42-Gal4*, and *Asap^B52/K23^; Letm1 RNA/D42-Gal4* mean values. These values relate to **Fig 4**.

| **Sl**  **No.** | **Genotypes** | **mEPSP**  **amplitude**  **(mV)** | **mEPSP**  **frequency**  **(Hz)** | **EPSP amplitude**  **(mV)** | **Quantal content**  **(QC)** | **GCaMP5G-td-Tomato ratio/µm² area of bouton** |
| --- | --- | --- | --- | --- | --- | --- |
| **1** | ***D42-Gal4/+*** | 0.83 ± 0.06 | 2.60 ± 0.30 | 37.32 ± 0.93 | 46.57 ± 3.58 | 100.0 ± 9.33 |
| **2** | ***D42-Gal4/plc-β* RNAi** | 0.75 ± 0.04 | 1.702 ± 0.09 | 36.77 ± 2.01 | 49.91 ± 4.85 | 80.34 ± 5.83 |
| **3** | ***Asap^K23/B52^*** | 0.79 ± 0.02 | 4.93 ± 0.26 | 45.86 ± 1.33 | 59.25 ± 3.10 | 160.44 ± 17.70 |
| **4** | ***Asap^K23/B52^; D42-Gal4/***  ***plc-β* RNAi** | 0.67 ± 0.02 | 3.30 ± 0.29 | 35.12± 2.24 | 56.65 ± 3.00 | 114.89 ± 5.52 |

**Table E.** The table shows values of mEPSP amplitude, mEPSP frequency, EPSP amplitude, Quantal content, and GCaMP5G-td-Tomato ratio of the above genotypes. The values represent mean±s.e.m. The control mean is compared with D*42-Gal4/plc-β* RNAi, *Asap^K23/B52^*, while the *Asap^K23/B52^* mean value is compared with the *Asap^K23/B52^; D42-Gal4/plc-β* RNAi animals. These values relate to **Fig 5**.

| **Sl**  **No.** | **Genotypes** | **# Boutons** | **Bouton area**  **(in µm^2^)** | **Inter-bouton**  **diameter**  **(in µm)** |
| --- | --- | --- | --- | --- |
| **1** | ***D42-Gal4/+*** | 18.20 ± 0.66 | 7.18 ± 0.51 | 0.98 ± 0.04 |
| **2** | ***Arf6^RNAi^/+; D42-Gal4/+*** | 20.50 ± 2.26 | 9.79 ± 0.19 | 1.24 ± 0.03 |
| **3** | ***D42-Gal4/Arf6^DN^*** | 20.62 ± 1.80 | 7.99 ± 0.77 | 0.91 ± 0.04 |
| **4** | ***D42-Gal4/Arf6^CA^*** | 12.50 ± 0.98 | 20.10 ± 0.81 | 1.54 ± 0.06 |
| **5** | ***Asap^B52/K23^*** | 13.60 ± 0.63 | 12.44 ± 0.33 | 1.68 ± 0.07 |
| **6** | ***Arf6^RNAi^, Asap^B52/K23^; D42-Gal4/+*** | 20.33 ± 0.77 | 9.57 ± 0.32 | 1.12 ± 0.10 |
| **7** | ***actin5C-Gal4, Asap^B52^/^K23^; UAS-Arf6^DN^/+*** | 20.28 ± 0.77 | 8.27 ± 0.679 | 0.98 ± 0.04 |
| **8** | ***Asap^B52/K23^; D42-Gal4/UAS-Arf6^DN^*** | 18.00 ± 1.00 | 6.46 ± 0.40 | 0.85 ± 0.04 |
| **9** | ***Asap^B52/K23^; mef2-Gal4/UAS-Arf6^DN^*** | 13.86 ± 0.82 | 12.12 ± 0.35 | 1.50 ± 0.04 |
| **10** | ***actin5C-Gal4, Asap^B52/K23^; UAS-Arf6^CA^/+*** | 13.85 ± 0.54 | 10.95 ± 0.39 | 1.42 ± 0.05 |
| **11** | ***Asap^B52/K23^; D42-Gal4/UAS-Arf6^CA^*** | 13.06 ± 0.58 | 10.69 ± 0.58 | 1.46 ± 0.04 |

**Table F.** The table shows values for average bouton number, average bouton area, and inter-bouton diameter on muscle 4 of A2 hemisegment in various genetic combinations as indicated. Values are represented as mean±s.e.m. These values relate to **Fig 6**.

| **Sl**  **No.** | **Genotypes** | **# of BRP puncta/μm^2^ area of bouton** | **Normalized BRP Intensity (A.U)/ μm^2^ area of bouton** | **Normalized Rab3 Intensity (A.U)/ μm^2^ area of bouton** |
| --- | --- | --- | --- | --- |
| **1** | ***D42-Gal4/+*** | 1.35 ± 0.04 | 100.0 ± 5.07 | 100.0 ± 6.69 |
| **2** | ***rab3^rup^/df*** | 0.48 ± 0.03 | 126.0 ± 5.50 | 7.40 ± 33.69 |
| **3** | ***D42-Gal4/Arf6^CA^*** | 1.85 ± 0.11 | 83.26 ± 3.14 | 245.9 ± 25.08 |
| **4** | ***rab3^rup^/df; D42-Gal4/Arf6^CA^*** | 0.59 ± 0.04 | 82.04 ± 3.91 | -10.50 ± 5.20 |
| **5** | ***Arf6 RNAi/+; D42-Gal4/+*** | 1.27 ± 0.05 | 163.47 ± 7.00 | 210.08 ± 7.60 |
| **6** | ***Arf6 RNAi,df/rab3^rup^; D42-Gal4/+*** | 0.53 ± 0.02 | 144.7 ± 10.44 | 1.80 ± 2.22 |

**Table G.** The table shows values for # of BRP puncta/μm^2^ area of bouton, normalized BRP intensity (A.U)/ μm^2^ area of bouton, and normalized Rab3 intensity (A.U)/ μm^2^ area of bouton in various genetic combinations as indicated. The control mean is compared with the mutant, while the mutant mean is compared with other combinations as indicated. Values are represented as mean±s.e.m. These values relate to **Fig 7**.

| **Sl**  **No.** | **Genotypes** | | | **% GCaMP5G/td-Tomato ratio (A.U)/µm^2^ area of bouton** | | | |
| --- | --- | --- | --- | --- | --- | --- | --- |
| **1** | ***OK371-Gal4/+; UAS-GCaMP5G-td-Tomato/+*** | | | 100.0 ± 7.16 | | | |
| **2** | ***OK371-Gal4/UAS-Arf6 RNAi; UAS-GCaMP5G-td-Tomato/+*** | | | 86.70 ± 4.99 | | | |
| **3** | ***OK6-Gal4, Asap^K23/B52^; UAS-GCaMP5G-td-Tomato/+*** | | | 165.78 ± 13.11 | | | |
| **4** | ***Arf6^RNAi^, Asap^B52^ /OK6-Gal4, Asap^K23^; UAS-GCaMP5G-td-Tomato/D42-Gal4*** | | | 119.17 ± 7.54 | | | |
| **Sl**  **No.** | **Genotypes** | **# of failures/**  **100 events** | **mEPSP**  **amplitude**  **(mV)** | | **mEPSP freq.**  **(Hz)** | **EPSP**  **amplitude**  **(mV)** | **Quantal**  **content**  **(QC)** |
| **1** | ***D42-Gal4/+*** | 28.25 ± 7.90 | 0.64 ± 0.03 | | 1.02 ± 0.07 | 43.73 ± 1.41 | 70.62 ± 5.30 |
| **2** | ***Asap^B52/K23^*** | 2.00 ± 1.42 | 0.77 ± 0.05 | | 2.91 ± 0.18 | 54.79 ± 1.26 | 74.87 ± 5.15 |
| **3** | ***D42-Gal4/***  ***UAS-Arf6^DN^*** | 0.08 ± 0.08 | 0.63 ± 0.02 | | 1.41 ± 0.15 | 42.06 ± 0.78 | 66.63 ± 2.76 |
| **4** | ***Asap^B52/K23^;***  ***UAS-Ar6^DN^/***  ***D42-Gal4*** | 21.36 ± 6.16 | 0.57 ± 0.05 | | 1.92 ± 0.26 | 44.44 ± 1.59 | 82.21 ± 9.84 |
| **5** | ***Arf6^RNAi^*/+*;***  ***D42-Gal4/+*** | 8.417 ± 8.32 | 0.60 ± 0.05 | | 1.55 ± 0.21 | 42.19 ± 1.23 | 73.29 ± 5.32 |
| **6** | ***Arf6^RNAi^,***  ***Asap^B52/K23^;***  ***D42-Gal4/+*** | 16.00 ± 4.94 | 0.77 ± 0.04 | | 1.94 ± 0.22 | 38.10 ± 1.53 | 49.98 ± 2.75 |

**Table H.** The table shows values of % of GCaMP5G/td-Tomato ratio (A.U.)/µm^2^ area of bouton, synaptic failures, mEPSP amplitude, mEPSP frequency, EPSP amplitude, and Quantal content in the indicated genotypes. Values are represented as mean±s.e.m. The control mean is compared with the mutant, while the mutant mean is compared with other combinations as indicated. These values relate to **Fig 8**.

| **Sl**  **No.** | **Genotypes** | **# Boutons** | **Bouton area**  **(in µm^2^)** | **Inter-bouton**  **diameter**  **(in µm)** |  |
| --- | --- | --- | --- | --- | --- |
| **1** | ***OK371-Gal4/+; D42-Gal4/+*** | 28.40 ± 1.29 | 8.75 ± 0.71 | 0.90 ± 0.03 |  |
| **2** | ***D42-Gal4/Arf6^CA^*** | 18.00 ± 1.32 | 15.10 ± 0.93 | 1.27 ± 0.06 |  |
| **3** | ***OK371-Gal4/plc-β RNAi*** | 22.30 ± 1.57 | 9.85 ± 0.80 | 0.80 ± 0.04 |  |
| **4** | ***OK371-Gal4/plc-β RNAi; D42-Gal4/Arf6^CA^*** | 19.30 ± 1.13 | 10.79 ± 0.75 | 1.14 ± 0.06 |  |
| **5** | ***OK371-Gal4/itpr* RNAi** | 19.70 ± 1.35 | 11.80 ± 0.98 | 0.93 ± 0.05 |  |
| **6** | ***OK371-Gal4/itpr RNAi; D42-Gal4/Arf6^CA^*** | 16.20 ± 1.28 | 10.77 ± 0.79 | 1.00 ± 0.05 |  |
| **7** | ***D42-Gal4/UAS-IP_3_-sponge*** | 18.10 ± 1.74 | 12.96 ± 0.98 | 0.94 ± 0.05 |  |
| **8** | ***OK371-Gal4/+; UAS-IP_3_-sponge, Arf6^CA^/D42-Gal4*** | 19.50 ± 1.40 | 10.94 ± 0.82 | 0.87 ± 0.04 |  |
| **Sl**  **No.** | **Genotypes** | **mEPSP**  **amplitude**  **(mV)** | **mEPSP**  **frequency**  **(Hz)** | **EPSP amplitude**  **(mV)** | **Quantal content**  **(QC)** |
| **1** | ***OK371-Gal4/+; D42-Gal4/+*** | 0.70 ± 0.06 | 2.29 ± 0.47 | 40.69 ± 2.41 | 59.72 ± 4.64 |
| **2** | ***D42-Gal4/Arf6^CA^*** | 0.64 ± 0.050 | 3.92 ± 0.64 | 46.70 ± 1.25 | 74.59 ± 5.23 |
| **3** | ***OK371-Gal4/plc-β RNAi*** | 0.67 ± 0.04 | 1.30 ± 0.19 | 43.95 ± 1.83 | 67.26 ± 4.87 |
| **4** | ***OK371-Gal4/plc-β RNAi; D42-Gal4/Arf6^CA^*** | 0.47 ± 0.02 | 2.74 ± 0.35 | 37.34 ± 1.92 | 80.47 ± 6.06 |
| **5** | ***OK371-Gal4/***  ***itpr* RNAi** | 0.72 ± 0.05 | 1.42 ± 0.38 | 32.95 ± 2.21 | 45.81 ± 1.76 |
| **6** | ***OK371-Gal4/itpr RNAi; D42-Gal4/Arf6^CA^*** | 0.72 ± 0.04 | 1.58 ± 0.21 | 37.23 ± 1.78 | 52.45 ± 2.76 |
| **7** | ***D42-Gal4*+Dantrolene (20µM)** | 0.66 ± 0.04 | 1.73 ± 0.29 | 41.12 ± 1.53 | 64.03 ± 4.64 |
| **8** | ***D42-Gal4/Arf6^CA^*+Dantrolene (20µM)** | 0.70 ± 0.03 | 2.52 ± 0.30 | 37.30 ± 1.62 | 53.87 ± 3.65 |
| **9** | ***D42-Gal4/UAS-IP_3_-sponge*** | 0.54 ± 0.02 | 1.91 ± 0.2807 | 39.52 ± 1.15 | 72.95 ± 2.89 |
| **10** | ***OK371-Gal4/+; UAS-IP_3_-sponge, Arf6^CA^/D42-Gal4*** | 0.64 ± 0.01 | 2.31 ± 0.2568 | 37.88 ± 1.52 | 58.95 ± 2.61 |

**Table I.** The table shows values of average bouton number, average bouton area, and inter bouton diameter on muscle 4 of A2 hemisegment, mEPSP amplitude, mEPSP frequency, EPSP amplitude and Quantal content in the indicated genotypes. Values are represented as mean±s.e.m. The control mean is compared with the mutant, while the mutant mean is compared with other combinations as indicated. These values relate to **Fig 9**.

| **Sl**  **No.** | **Genotypes** | **# Boutons** | **Bouton area**  **(in µm^2^)** | **Bouton size**  **(in µm)** | **Inter-bouton**  **diameter**  **(in µm)** |
| --- | --- | --- | --- | --- | --- |
| **1** | ***w^1118^* (Control)** | 18.00 ± 1.12 | 11.30 ± 0.37 | 3.55 ± 0.05 | 0.92 ± 0.06 |
| **2** | ***Asap^K23^*** | 12.86 ± 0.82 | 14.31 ± 0.67 | 4.09 ± 0.09 | 1.73 ± 0.03 |
| **3** | ***Asap^B52^*** | 13.06 ± 0.48 | 16.48 ± 0.58 | 4.47 ± 0.09 | 1.65 ± 0.06 |
| **4** | ***Asap^B52/K23^*** | 9.86 ± 0.38 | 16.56 ± 0.71 | 4.41 ± 0.09 | 1.92 ± 0.03 |

**Table J.** The table shows the analysis of total bouton number, average bouton area, average bouton size, and average inter-bouton bouton diameter on muscle 4 of A2 hemisegment in the indicated genotypes above. Error bars represent mean±s.e.m. The control mean is compared with the mutants as indicated. These values relate to **S1 Fig**.

| **Sl**  **No.** | **Genotypes** | **# Boutons** | **Bouton area**  **(in µm^2^)** | **Inter-bouton**  **diameter**  **(in µm)** | **mEPSP**  **amplitude**  **(mV)** | **mEPSP**  **frequency**  **(Hz)** | **EPSP amplitude**  **(mV)** | **Quantal content**  **(QC)** |
| --- | --- | --- | --- | --- | --- | --- | --- | --- |
| **1** | ***D42-Gal4/+*** | 26.62±2.64 | 7.92 ± 0.47 | 0.81±0.02 | 0.82 ± 0.03 | 1.74 ± 0.23 | 46.39±1.42 | 57.34±3.83 |
| **2** | ***Asap^B52/K23^; D42-Gal4/+*** | 12.75±0.72 | 14.31±0.89 | 1.80±0.10 | 1.03 ± 0.06 | 4.28 ± 0.38 | 55.93±1.50 | 55.98±3.93 |
| **3** | ***Asap^B52/K23^; UAS-Asap^FL^/+*** | 12.12±1.12 | 13.96±0.57 | 1.57±0.10 | 0.89 ± 0.08 | 4.53 ± 0.56 | 54.70±0.41 | 64.79±6.00 |

**Table K.** The table shows values for average bouton number, average bouton area, and interbouton diameter on muscle 4 of A2 hemisegment, in various genetic combinations as indicated. Values are represented as mean±s.e.m. The control mean is compared with the mutant. These values relate to **S3 Fig**.

| **Sl**  **No.** | **Genotypes** | **% of microtubule loops** | **# of Moesin GFP puncta/ µm^2^ area of NMJ** | **# of Actin-GFP puncta/ µm^2^ area of NMJ** | **Relative Ac-tubulin intensity/ µm^2^ area of NMJ** | **Relative α-tubulin levels** | **Relative Ac-tubulin levels** |
| --- | --- | --- | --- | --- | --- | --- | --- |
| **1** | ***w^1118^* (Control)** | 52.14 ± 2.84 | 0.18 ± 0.01 | 0.17 ± 0.01 | 100.0 ± 4.49 | 1.0 ± 0.0 | 1.0 ± 0.0 |
| **2** | ***Asap^B52/K23^*** | 19.47 ± 2.23 | 0.19 ± 0.02 | 0.19 ± 0.02 | 84.30 ± 5.81 | 1.16 ± 0.08 | 1.04 ± 0.11 |
| **3** | ***Asap^B52/K23^; UAS-***  ***Asap^FL^/D42-Gal4*** | 48.96 ± 3.73 | - | - | 84.44 ± 5.92 | 1.10 ± 0.11 | 0.99 ± 0.11 |

**Table L.** The table shows the analysis of % of microtubule loops, the number of Moesin GFP puncta, the number of Actin-GFP puncta, the relative Ac-tubulin intensity, the relative α-tubulin, and the Ac-tubulin levels in the indicated genotypes. Error bars represent mean±s.e.m. The control mean is compared with the mutants as indicated. These values relate to **S4 Fig**.

| **Sl**  **No.** | **Genotypes** | **Paired pulse ratio**  **(0.1 mM Ca^2+^)** | **Paired pulse ratio**  **(0.4 mM Ca^2+^)** | **Paired pulse ratio**  **(1.5 mM Ca^2+^)** | **EPSP amplitude**  **(0.1 mM Ca^2+^)** |
| --- | --- | --- | --- | --- | --- |
| **1** | ***D42-Gal4/+* (Control)** | 1.20 ± 0.15 | 0.98 ± 0.02 | 0.86 ± 0.04 | 6.43 ± 1.32 |
| **2** | ***Asap^B52/K23^*** | 0.92 ± 0.04 | 0.90 ± 0.00 | 0.83 ± 0.02 | 19.91 ± 3.44 |
| **3** | ***Asap^B52/K23^; UAS-***  ***Asap^FL^/D42-Gal4*** | 1.06 ± 0.10 | 0.92 ± 0.02 | 0.87 ± 0.01 | 9.80 ± 2.38 |
| **4** | ***Asap^B52/K23^;D42-Gal4/UAS-Arf6^DN^*** | 1.11 ± 0.12 | 0.95 ± 0.02 | 0.85 ± 0.01 | 10.98 ± 2.38 |

**Table M.** The table shows the analysis of paired pulse ratio and EPSP amplitudes at 0.1, 0.4, 1.5, and 0.1 mM Ca^2+^ concentrations in the indicated genotypes. Error bars represent mean±s.e.m. The control mean is compared with the mutant mean, and the mutant mean is compared with the *Asap* rescue and *Arf6^DN^* rescue combinations. These values relate to **S5 Fig**.

| **Sl**  **No.** | **Genotypes** | **% GCaMP5G/td-Tomato ratio (A.U)/μm2 area of bouton** |
| --- | --- | --- |
| **1** | ***OK371-Gal4/+; UAS-GCaMP5G-td-Tomato/+* (Unstimulated)** | 100.0 ± 4.39 |
| **2** | ***OK371-Gal4/+; UAS-GCaMP5G-td-Tomato/+* (Stimulated, 90 mM KCl)** | 130.8 ± 16.73 |
| **3** | ***OK371-Gal4, Asap^B52^/^K23^; UAS-GCaMP5G-td-Tomato/+* (Unstimulated)** | 100.0 ± 6.00 |
| **4** | ***OK371-Gal4, Asap^B52^/^K23^; UAS-GCaMP5G-td-Tomato/+* (Stimulated, 90 mM KCl)** | 204.7 ± 33.81 |

**Table N.** The table shows values for % GCaMP5G/td-Tomato ratio (A.U)/μm2 area of bouton in the indicated genotypes. Values are represented as mean±s.e.m. These values relate to **S6 Fig**.

| **Sl**  **No.** | **Genotypes** | **% GCaMP5G/td-Tomato ratio**  **(A.U)/μm^2^ area of bouton** |
| --- | --- | --- |
| **1** | ***OK-371-Gal4/+; UAS-GCaMP5G-td-Tomato/+*** | 100.0 ± 7.78 |
| **2** | ***OK371-Gal4, Asap^B52/K23^; UAS-GCaMP5G-td-Tomato/+*** | 182.4 ± 13.23 |
| **3** | ***OK371-Gal4/+; UAS-GCaMP5G-td-Tomato/itpr RNAi*** | 41.72 ± 3.90 |
| **4** | ***OK371-Gal4, Asap^B52^/OK371-Gal4, Asap^K23^; UAS-GCaMP5G-td-Tomato/itpr RNAi*** | 115.84 ± 7.39 |
| **5** | ***OK371-Gal4/+; UAS-GCaMP5G-td-Tomato/ RyR RNAi*** | 74.39 ± 12.11 |
| **6** | ***OK371-Gal4, Asap^B52^/OK371-Gal4, Asap^K23^; UAS-GCaMP5G-td-Tomato/RyR RNAi*** | 59.18 ± 3.68 |
| **7** | ***OK-371-Gal4/+; UAS-GCaMP5G-td-Tomato/+*Dantrolene** | 45.50 ± 6.03 |
| **8** | ***OK371-Gal4, Asap^B52^/OK371-Gal4, Asap^K23^; UAS-GCaMP5G-td-Tomato/itpr RNAi*+ Dantrolene** | 83.77 ± 5.87 |

**Table O.** The table shows values for % of GCaMP5G/td-Tomato ratio (A.U.)/µm^2^ area of bouton in various genetic combinations as indicated. Values are represented as mean±s.e.m. The control mean is compared with the mutant, while the mutant mean is compared with other combinations as indicated. These values relate to the **S7 Fig**.

| **SL**  **NO** | **Genotypes** | **mEPSP**  **amplitude**  **(mV)** | **mEPSP**  **frequency**  **(Hz)** | **EPSP amplitude**  **(mV)** |
| --- | --- | --- | --- | --- |
| 1 | ***w^1118^* (Control)** | 0.63 ± 0.04 | 0.92 ± 0.09 | 28.03 ± 1.71 |
| 2 | ***Asap^B52/K23^*** | 0.70 ± 0.04 | 4.11 ± 0.62 | 43.13 ± 1.88 |
| 3 | ***itpr^90B.0^/+*** | 0.66 ± 0.04 | 1.54 ± 0.27 | 29.72 ± 2.63 |
| 4 | ***Asap^B52/K23^; itpr ^90B.0^/+*** | 0.69 ± 0.07 | 2.77 ± 0.48 | 33.95 ± 3.56 |
| 5 | ***RyR^E4340K^/+*** | 0.54 ± 0.03 | 1.53 ± 0.24 | 36.97 ± 1.17 |
| 6 | ***Asap^B52^, RyR^E4340K^/Asap^K23^*** | 0.86 ± 0.07 | 2.82 ± 0.21 | 29.70 ± 2.59 |

**Table P.** The table shows values of mEPSP amplitude, EPSP amplitudes, and mEPSP frequency in the indicated genotypes. Values are represented as mean±s.e.m. The control mean is compared with the mutant, while the mutant mean is compared with other combinations as indicated. These values relate to **S8 Fig**.

| **SL**  **NO** | **Genotypes** | **% of Futsch**  **positive loops** | **# of BRP puncta/**  **NMJ** | **Normalized Rab3 Intensity (A.U)/ μm^2^ area of bouton** |
| --- | --- | --- | --- | --- |
| **1** | ***D42-Gal4/+* (Control)** | 32.59 ± 1.72 | 519.16 ± 37.44 | 100.0 ± 4.32 |
| **2** | ***Asap^B52/K23^*** | 17.88 ± 1.09 | 1053.16 ± 107.4 | 68.96 ± 4.40 |
| **3** | ***D42-Gal4/***  ***UAS-Arf6^DN^*** | 21.50 ± 1.47 | 265.92 ± 9.91 | 170.90 ± 8.12 |
| **4** | ***Asap^B52/K23^;***  ***UAS-Ar6^DN^/***  ***D42-Gal4*** | 27.25 ± 1.54 | 439.50 ± 41.86 | 125.85 ± 6.54 |
| **5** | ***Arf6^RNAi^*/+*;***  ***D42-Gal4/+*** | 20.50 ± 1.22 | 270.84 ± 11.60 | 158.70 ± 6.69 |
| **6** | ***Arf6^RNAi^,***  ***Asap^B52/K23^;***  ***D42-Gal4/+*** | 40.63 ± 1.03 | 607.62 ± 69.01 | 121.11 ± 6.32 |

**Table Q.** The table shows values of the percentage of Futsch-positive loops, the average number of Brp puncta per NMJ, and the normalized Rab3 intensity in the indicated genotypes. Values are represented as mean±s.e.m. The control mean is compared with the mutant, while the mutant mean is compared with other combinations as indicated. These values relate to **S9 Fig**.

| **SL**  **NO** | **Genotypes** | **Normalized BRP Intensity (A.U)/ μm^2^ area of bouton** | **# of BRP puncta/μm^2^ area of bouton** | **Normalized Rab3 Intensity (A.U)/ μm^2^ area of bouton** |
| --- | --- | --- | --- | --- |
| **1** | ***D42-Gal4/+*** | 100.0 ± 5.83 | 1.25 ± 0.04 | 100.0 ± 7.02 |
| **2** | ***Asap^B52/K23^*** | 73.48 ± 4.60 | 1.63 ± 0.05 | 65.50 ± 3.40 |
| **3** | ***D42-Gal4/plc-β RNAi*** | 128.76 ± 7.71 | 1.26 ± 0.05 | 217.37 ± 10.12 |
| **4** | ***Asap^B52/K23^*; *D42-Gal4/plc-β RNAi*** | 167.11 ± 10.54 | 1.52 ± 0.06 | 194.28 ± 15.85 |
| **5** | ***D42-Gal4/itpr* RNAi** | 58.21 ± 2.96 | 1.33± 0.08 | 168.26 ± 13.91 |
| **6** | ***Asap^B52/K23^*; *D42-Gal4/itpr RNAi*** | 195.13 ± 14.37 | 1.80 ± 0.07 | 188.07 ± 10.34 |
| **7** | ***D42-Gal4/RyR RNAi*** | 82.63 ± 3.08 | 1.62 ± 0.07 | 160.98 ± 8.55 |
| **8** | ***Asap^B52/K23^*; *D42-Gal4/RyR RNAi*** | 149.30 ± 8.13 | 2.01 ± 0.09 | 163.76 ± 7.05 |

**Table R.** The table shows values for normalized BRP intensity (A.U)/ μm^2^ area of bouton, # of BRP puncta/μm^2^ area of bouton, and normalized Rab3 intensity (A.U)/ μm^2^ area of bouton in various genetic combinations as indicated. The control mean is compared with the mutant, while the mutant mean is compared with other combinations as indicated. Values are represented as mean±s.e.m. These values relate to **S10 Fig**.

| **SL**  **NO** | **Genotypes** | **Relative transcript level** |
| --- | --- | --- |
| **1** | ***actin5C-Gal4/+*** | 1.00 ± 0.00 |
| **2** | ***Arf6 RNAi (V100726)*** | 0.52 ± 0.03 |
| **3** | ***actin5C-Gal4/+*** | 1.00 ± 0.00 |
| **4** | ***Plc21C RNAi (BL31269)*** | 0.42 ± 0.08 |
| **5** | ***Plc21C RNAi (V26557)*** | 0.40 ± 0.05 |
| **6** | ***actin5C-Gal4/+*** | 1.00 ± 0.00 |
| **7** | ***Letm1 RNAi (BL37502)*** | 0.64 ± 0.06 |
| **8** | ***actin5C-Gal4/+*** | 1.00 ± 0.00 |
| **9** | ***itpr RNAi (BL25937)*** | 0.62 ± 0.08 |
| **10** | ***itpr RNAi (BL51686)*** | 0.63 ± 0.07 |
| **11** | ***actin5C-Gal4/+*** | 1.00 ± 0.00 |
| **12** | ***RyR RNAi (BL29445)*** | 0.52 ± 0.07 |

**Table S.** The table shows values for relative transcript level in the indicated genotypes. The control mean is compared with the RNAi. Values are represented as mean±s.e.m. These values relate to **S11 Fig**.

| **Primer Name** | **Sequence (5′-3′)** |
| --- | --- |
| **PLC21C RT FP** | TTGAAGGAGCGACCGATTG |
| **PLC21C RT RP** | CAGAACAACTGCGGCATAAAG |
| **Itpr RT FP** | CTGCTGCAGGATATAGTGTACTT |
| **Itpr RT RP** | CCGATCTCTGATTGGGTTCTT |
| **Arf6 rt FP** | AATGTGGAAACCGTCACCTATAA |
| **Arf6 rt RP** | CCGTGTAATAGTGTCGCCATAG |
| **Letm1 rt FP** | GAGTGCTCAACGGCAAGA |
| **Letm1 rt RP** | ATGAAGGGCACGATGATGAA |
| **RyR rt FP** | GGATTTGATGGTGCCTTTCTTT |
| **RyR rt RP** | CGCCAATAACATCGCCTTTAC |

**Table T.** The table lists the primers used for the RT-qPCR.
