# Supplementary material for: An ArfGAP-dependent signaling modulates synaptic plasticity via IP3-regulated calcium release from the endoplasmic reticulum
Source: PLoS Genet. 2026 Jan 23;22(1):e1012031. doi: 10.1371/journal.pgen.1012031 (PMC12863683; doi:10.1371/journal.pgen.1012031)
Supplement: Attachment — Submitted filename: Rebuttal Plos Genetics R1_071125.docx [file pgen.1012031.s015.docx]

Please find below our detailed point-by-point responses to other reviewer's comments:

**Manuscript ID: PGENETICS-D-25-00364**

**Reviewer #1:**

*IP3 and ryanodine receptors are known to regulate synaptic strength. However, the upstream mechanisms that regulate IP3 and ryanodine receptors are not well understood. Mallik et al. investigated the role of Drosophila Asap in synapse development and calcium homeostasis. They found that loss of Asap resulted in structural changes (increased bouton size, reduced bouton number, and higher active zone density), increased mEJP frequency, reduced synaptic failures under low extracellular Ca2+, and elevated basal presynaptic calcium levels. Genetic epistatic experiments suggest that Asap regulates Arf6 and restricts calcium release through the IP3-dependent pathway. Overall, this is an interesting study that provides mechanistic insight into the upstream regulation of IP3 and ryanodine receptors that affects synaptic structure and function. Below are several points that I think would improve the manuscript.*

**Major Points**

***1. Supplemental Figure 2 indicates that the Asap antibodies are not specific for Asap protein, given that staining is observed in the Asap null (Fig. S2C). The fact that pre-absorption of antibody with pure Asap protein eliminated all staining doesn't mean that any of the staining was Asap protein. In Fig. 1G, the authors performed Western blots on fillet preparations and detected Asap protein in the mef2-GAL4 rescue line. If the authors ran a Western on control larval fillet preps and also detected Asap protein, this would suggest that Asap is likely expressed at the NMJ.***

**Response:** We appreciate the reviewer's insightful comment. As indicated in Figure 1G, we have already conducted a Western blot analysis using control larval fillet preparations. The results show a band at the expected molecular weight of approximately 128 kDa, corresponding to the Asap protein, confirming the presence of the Asap protein in the control samples. This finding supports that Asap is endogenously expressed at the neuromuscular junction (NMJ). We even generated peptide antibodies against Asap, but these antibodies also could not eliminate the background staining in the *Asap* mutants (data not shown), suggesting the presence of a very similar protein(s) at the NMJ.

***2. How was GluRII cluster area calculated?***

**Response:** The GluRIII cluster area at the larval neuromuscular junction was quantified from confocal images by identifying GluRIII puncta that were apposed to active zone puncta. A binary mask was generated in ImageJ to delineate the boundaries of each GluRIII cluster, and the corresponding area was measured in square micrometers (µm²). These methodological details have been clarified and updated in the **revised Materials and Methods section**.

***3. Increased basal presynaptic calcium levels likely explain the increased mEJP frequency seen in Asap mutants. Did the authors look at presynaptic calcium levels in response to stimulation to determine if changes in presynaptic calcium were responsible for the increased EJP amplitudes? This is important in linking changes in evoked neurotransmission to changes in presynaptic calcium. This would be a better experiment than doing electrophysiology in the presence of BAPTA-AM.***

**Response:** We thank the reviewer for the insightful comment regarding the relationship between presynaptic calcium dynamics and enhanced evoked neurotransmission in *Asap* mutants. Our findings indicate that basal presynaptic calcium levels are already elevated in *Asap* mutants, likely contributing to

the increased mEJP frequency. Although presynaptic calcium transients during stimulation were not directly measured, it is reasonable to expect that repetitive stimulation would further amplify neurotransmitter release in these mutants. Previous studies have demonstrated that repetitive stimulation enhances transmitter release through distinct calcium-dependent mechanisms, including facilitation, augmentation, and potentiation [1]. Consistent with this, experimental manipulations that elevate intracellular calcium potentiate evoked release by enhancing calcium-dependent components of synaptic transmission. Therefore, the increased EJP amplitudes observed in *Asap* mutants likely result from altered presynaptic calcium handling. Future experiments involving pulsed stimulation coupled with GCaMP imaging will further strengthen the link between presynaptic calcium dynamics and the enhanced evoked neurotransmission in *Asap* mutants. However, our data supports that a combination of elevated basal calcium and increased active-zone numbers (as estimated from increased Brp number and density) likely results in increased EPSP in *Asap* mutants.

***4. What concentration of BAPTA-AM was used in Fig. 4? It's surprising that BAPTA-AM did not reduce evoked neurotransmission in controls.***

**Response:** BAPTA-AM is a fast calcium chelator commonly used to buffer intracellular Ca²⁺. Previous studies have reported reductions in miniature frequency following 25–30 minutes of incubation [2, 3]. In the experiments shown in Figure 4, BAPTA-AM was applied at 20 µM, and preparations were incubated for 25–30 minutes and washed before electrophysiological recordings. Consistent with our previous findings [3, 4], this treatment did not significantly reduce evoked neurotransmission in control NMJs, suggesting that the basal calcium-buffering capacity in control terminals remains sufficient to maintain normal evoked release under these experimental conditions.

***5. It would be good to confirm that UAS and GAL4 controls do not rescue Asap phenotypes in Fig. 4, 5, 6, and 7. There could be insertional effects or leaky expression from UAS lines.***

**Response:** We thank the reviewer for this insightful suggestion. To address potential insertional effects or leaky expression, we examined UAS-transgene and GAL4 driver lines individually in the *Asap* mutant background. Both genotypes exhibited NMJ morphology and electrophysiological properties comparable to those of *Asap* mutants, indicating that neither the GAL4 driver nor the UAS transgene alone rescues the mutant phenotype. These results confirm that the observed phenotypes are specific to *Asap* function and not due to background or insertional artifacts. The corresponding data are included in **Supplemental Figure S3.**

***6. Also, have Ryanodine, Letm1, PLCB, and Arf6 RNAi lines been verified to show that they do knock down Ryanodine, Letm1, PLCB, and Arf6, respectively?***

**Response:** We have now verified the efficiency of RNAi-mediated knockdown of *RyR*, *itpr*, *Letm1*, *Arf6,* and *Plc21C* using quantitative real-time PCR. Our results demonstrate a significant reduction in the corresponding mRNA transcript levels, confirming the effective silencing of the gene. These validation data are provided in **Supplemental Figure 10**.

**Reviewer #2:**

This manuscript uncovers a potentially interesting role of ArfGAP in synaptic development and neurotransmission. While the authors provide a substantial amount of data, several critical issues need to be addressed to strengthen the conclusions:

**Major Points**

1. ***The genotypes of the control groups in each Figure are not clearly indicated. This information is essential for interpreting the experimental results.***

**Response:** We appreciate the reviewer's insightful comment. The genotypes of the control groups used in each experiment are now clearly indicated in the Figure and are written in the legend section.

***2. There are notable inconsistencies in mEPSP, EPSP amplitudes, and quantal content (QC) across Figures 2, 3, 4, 5 and 7. Are these differences due to varying extracellular Ca²***⁺ ***concentrations or genetic backgrounds? Such methodological details must be clearly stated.***

**Response:** The observed variations in mEPSP, EPSP amplitudes, and quantal content (QC) across Figures 2, 3, 4, 5, and 7 primarily reflect differences in extracellular calcium concentrations (0.1, 0.5, and 1.5 mM) and the use of distinct genetic backgrounds (*w^1118^* or Gal4 driver controls). These experimental parameters are specified in the Methods section and corresponding figure legends to ensure accurate interpretation of the data.

***3. Concerns regarding Figure 4:***

***The identities and descriptions of panels 4O and 4P are missing from the figure legend and main text. The data suggest that loss of Asap increases resting Ca²***⁺ ***levels and slows calcium dynamics. In this context, BAPTA-AM, which preferentially chelates fast calcium transients, may not be appropriate. EDTA-AM would be more suitable for buffering slow Ca²***⁺ ***changes. BAPTA-AM reduces EPSP amplitude in the Asap mutant; however, it is unclear why a similar effect is not observed in the control. Since RNAi knockdown of itpr and RyR already reduces mEPSP frequency in the control, it is unclear whether these ER calcium channels are solely responsible for the elevated mEPSP frequency in Asap mutants. Testing these effects in heterozygous itpr and RyR mutant backgrounds would help clarify this. Does knockdown of iptr or RyR also normalize resting Ca²***⁺ ***levels in the Asap mutant? This is important data. A double knockdown condition (2xD42>itpr-RNAi + RyR-RNAi) should be included. The effect of Letm1 knockdown in the control background is missing, making it difficult to interpret its role.***

**Response:** We thank the reviewers for highlighting these points. Panels 4O and 4P, corresponding to Control+DMSO and *Asap* mutant+DMSO, the DMSO is mistakenly labelled; this has now been removed, described, and incorporated into both the figure legends and the main text.

We acknowledge that BAPTA-AM is a fast calcium chelator, typically used to buffer rapid intracellular Ca²⁺ transients. Consistent with prior reports [2, 4], 25-30 minutes of BAPTA-AM incubation reduced miniature frequency without significantly affecting evoked neurotransmission in control NMJs. These findings indicate that basal calcium-buffering capacity in control terminals is sufficient to maintain normal evoked release under these experimental conditions.

As recommended by the reviewer, we additionally evaluated the influence of slower calcium chelation using EDTA-AM. Similar to BAPTA-AM, EDTA-AM treatment had no measurable effect on EPSP amplitude or miniature frequency in control NMJs. In *Asap* mutants, however, where intracellular Ca²⁺ levels are elevated, the slow-binding kinetics of EDTA-AM resulted in a modest reduction in EPSP amplitude, consistent with increased sensitivity of evoked release to intracellular calcium perturbation. Together, these findings support the conclusion that excessive intracellular calcium in *Asap* mutants contributes to both the elevated spontaneous release and the reduced evoked neurotransmission observed at their NMJs.

The corresponding data is enclosed below for the reviewer's perusal:

**Figure: Low-affinity calcium chelation by EDTA-AM partially restores the EPSP in *Asap* mutants**

(**A-D**) Representative traces of mEPSP and EPSP in (A) *w^1118^* +DMSO, Wash (B) *w^1118^* +EDTA-AM, Wash , *Asap^B52/K23^*+DMSO, Wash and (D) *Asap^B52/^*^K2^+EDTA-AM,Wash preparations. Scale bars for EPSPs (mEPSP) are x = 50 ms (1000 ms) and y = 10 mV (1 mV).

(**E-H**) Histogram showing mEPSP amplitude (E), mEPSP frequency (F), EPSP amplitude (G) and Quantal content (H) from muscle 6 of A2 hemisegment in the indicated genotypes. **p*=0.01 (EPSP amplitudes). The error bar represents the standard error of the mean (SEM). The statistical analysis was performed using a Student's t-test; ns indicates not significant. n=7-10 NMJ per genotype. The values for each quantification are shown in Supplemental Table S16. All recordings included in the analysis have an input resistance greater than 5 MΩ.

To assess whether ER calcium channels are solely responsible for the elevated mEPSP frequency in *Asap* mutants, we examined *Asap* mutants in heterozygous *itpr* and *RyR* mutant backgrounds. We observed reductions in both mEPSP frequency and EPSP amplitude in these heterozygous combinations compared with *Asap* mutants alone (**Supplemental Figure 7).** Furthermore, calcium imaging in *Asap* mutant synapses following *iptr* or *RyR* knockdown, as well as combined *iptr* inhibition in *RyR* RNAi backgrounds, revealed decreased calcium signals relative to *Asap* mutants, indicating restoration of calcium homeostasis (**Supplemental Figure 6)**.

Finally, the effects of *Letm1* knockdown in control backgrounds have now been assessed and incorporated into **Figure 4U and 4V**, providing additional context for its role in calcium dynamics and synaptic function.

***4. Concerns regarding Figure 5***

***Knockdown of plcβ reduces mEPSP frequency similarly in both control and Asap mutants, casting doubt on its proposed role in mediating the Asap-dependent increase in miniature events. Furthermore, no data on resting Ca²***⁺ ***levels are provided in this Figure.***

**Response:** To support our conclusion that PLCβ mediates Asap-dependent increase in miniature events, we have now measured calcium signals in *Asap* mutant NMJs following *Plc21C* knockdown. These experiments reveal a partial restoration of calcium dynamics compared to *Asap* mutants alone. The corresponding data have been incorporated into the revised **Figure 5**.

***5. Concerns regarding Figure 6***

***The effect of Arf-RNAi, Arf6-DN, or Arf6-CA expression alone on bouton morphology is not shown. Without these controls, it is difficult to conclude whether increased Arf activity is causally related to the bouton phenotype in Asap mutants.***

**Response:** We thank the reviewer for this suggestion. We have now performed these experiments, and the following data are incorporated in **Figure 6**. We found that while motor neuron-driven Arf6 RNAi or Arf6^DN^ did not show a significant change in NMJ morphology, expression of constitutively active Arf6^CA^ phenocopied Asap mutations. This data further supports our conclusion that Asap functions through active Arf6 to regulate synaptic morphogenesis.

***6. Concerns regarding Figure 7***

***As in Figure 6, the effects of Arf6-RNAi expression alone should be presented. It is unclear whether Arf6-DN expression also reduces the elevated resting Ca²***⁺ ***levels in Asap mutants.***

**Response:** We thank the reviewer for this suggestion. We have now incorporated physiological data for D42-Gal4>Arf6RNAi and D42-Gal4>Arf6DN, as well as calcium imaging data for D42-Gal4>Arf6 RNAi, corresponding to the experiments in **Figure 8**. Because both GCaMP5G and Arf6-DN are GFP-tagged, we employed an untagged Arf6-RNAi construct to circumvent fluorescence overlap, thereby allowing for the accurate measurement of calcium signals in *Asap* mutant backgrounds.

***7. It is not investigated whether active zone intensity and VGCC activity may contribute to the phenotype of the Asap mutant. Since Asap mutants show increased active zone intensity, it is possible that this change enhances VGCC activity and contributes to the elevated resting Ca²***⁺ ***concentration. The authors should test whether knockdown of IP3R, RyR, Arf6, or Arf6-DN also rescues this active zone phenotype.***

**Response:** We appreciate the reviewer's important comment. To address the potential contribution of active zone intensity and VGCC activity to the *Asap* mutant phenotype, we measured both Brp and CAC (Cacophony) intensities in control, *Asap* mutants, and rescue combinations. These new data reveal an approximate 45% decrease in Brp intensity and ~50% decrease in CAC intensity in *Asap* mutants. In contrast, AZ density, assessed as Brp and CAC puncta per NMJ, is concurrently increased compared with controls. These findings align with prior studies showing that changes in Brp intensity and density can be inversely regulated as part of homeostatic active zone dynamics [4, 5]

To determine whether modulation of intracellular calcium signaling could rescue the AZ phenotype observed in *Asap* mutants, we knocked down *itpr*, *plcβ*, and *RyR* in the *Asap* mutant background. These experiments yielded mixed outcomes. For instance, PLCβ knockdown showed more Brp intensity while knockdown of itpr reduced the Brp intensity without affecting the level of Rab3 (**Supplemental S9**), suggesting that multiple independent and potentially combinatorial processes

regulated IP3 signaling may converge on active zone organization. This complexity warrants further investigation to delineate the specific contributions of ER-derived calcium signaling in the regulation of synaptic organization. This incomplete suppression is likely attributable to the partial knockdown efficiency of the RNAi lines, which reduced transcript levels by approximately 40–50% (**Supplemental Figure 10**). Knockdown of Arf6 or expression of Arf6^DN^ in the Asap mutant background rescued the Brp density and number (**Supplemental Figure 8**). Collectively, these results suggest that active zone organization in *Asap* mutants is partly modulated by Arf6-dependent pathways, and that a partial reduction in IP3R or RyR is insufficient to fully rescue AZ density.

***8. It is unclear whether microtubules play a potential role in ER channel regulation. Given the established link between ER organization and microtubule dynamics, it is plausible that altered microtubule architecture underlies the observed increase in ER Ca²***⁺ ***channel activity. This possibility should be explored.***

**Response:** We agree with the reviewer that microtubules may play a role in regulating ER Ca²⁺ channels. In *Asap* mutants, we observe destabilized microtubules alongside increased ER Ca²⁺ release, consistent with prior studies showing that microtubule stability influences ER morphology and Ca²⁺ handling (e.g., *Spastin* mutants; Microtubule stabilization by mutant Spastin affects ER morphology and Ca²⁺ handling [6]. Hence, it is likely that microtubule destabilization in *Asap* mutants may disrupt ER architecture or the spatial positioning of ER Ca²⁺ channels, thereby enhancing their activity. Future experiments assessing microtubule-ER interactions and ER channel localization will be important to directly test this mechanistic link. We have added this possibility in the discussion explaining the role of microtubules in regulating the ER channel.

***9. The authors claim that actin and moesin structures are unaltered in Asap mutants (Supplemental Figure S3), yet only acetylated tubulin staining is shown. This statement should be revised accordingly.***

**Response:** We thank the reviewer for pointing this out. In our study, western blot analysis showed no changes in total tubulin or acetylated tubulin levels in *Asap* mutants **(Figure S4F–H).** However, immunostaining in **Supplemental Figure S4A–C, E** only depicts acetylated tubulin. Based on these data, we conclude that both total and acetylated tubulin are unaltered in *Asap* mutants. We have revised the relevant statements in the main text to accurately reflect these findings.

***10. The abstract refers to "pharmacological and genetic manipulations," yet no pharmacological experiments are included. This statement should be corrected.***

**Response:** We thank the reviewer for this observation. In the revised manuscript, we have included experiments using pharmacological agents BAPTA-AM, EDTA-AM, and Dantrolene in NMJ preparations. Accordingly, we believe it is now appropriate to refer to both pharmacological and genetic manipulations in the abstract.

***11. The error bars in Figure 3 appear to represent standard deviation but are labeled as SEM. This should be corrected for consistency and clarity.***

**Response:** We thank the reviewer for bringing this to our attention. We revisited the raw data and modified it as suggested.

**Reviewer #3:**

The authors examine the role of Arf-specific GAP Asap in modulating IP3-regulated calcium release from the ER at the *Drosophila* NMJ. They generated *Asap* mutants and other genetic reagents to perform phenotypic and quantitative analyses in different morphological and electrophysiological assays. The authors show that loss of *Asap* elevates resting calcium in synaptic terminals, enhances vesicle release, and disrupts both synaptic morphology and microtubule organization. Further genetic interaction

experiments with PLCβ and Arf6 suggest a novel mechanism in which Asap inhibits Arf6-PLCβ-IP3 signaling via its ArfGAP activity, thereby controlling calcium release from the ER.

***The work presented is interesting and I particularly enjoy the extensiveness of the assays the authors employed. Overall, the phenotypic characterization of Asap mutants using morphological markers and electrophysiological assays is quite extensive. However, the evidence of functional link between Asap and Arf6 or PLCβ is rather weak. In Fig.5 F-I, PLCβ RNAi itself is not statistically different from control, raising the possibility of a compensatory effect of PLCβ RNAi upon loss of Asap. Moreover, in Fig.6-7 and Fig.S4-5, the experiments were done for Arf6 DN in Asap mutant background, but no data for Arf6 mutants or Arf6 DN alone. Therefore, the authors' conclusion regarding the Asap-dArf6 regulatory relationship extends beyond what the current data can substantiate.***

***Another criticism is the lack of proper genetic controls throughout the manuscript. The authors used the Gal4/UAS system extensively but seemed not to consider the controls for these manipulations. For instance, in the rescue experiments at least UAS-Asap alone, i.e. without the Gal4 drivers, should be performed as a proper control. For RNAi or overexpression of Arf6-DN, Gal4 alone and UAS-RNAi or UAS-Arf6-DN should be performed.***

**Response:** We thank the reviewer for these valuable comments and for highlighting the importance of appropriate controls and functional validation. To strengthen our conclusions regarding the link between Asap, Arf6, and PLCβ (Plc21C), we have expanded our analyses and incorporated additional data.

Our quantitative real-time PCR analysis revealed approximately a ~55% reduction in *Plc21C* transcript levels under RNAi knockdown conditions (**Supplemental Figure 10**). This partial knockdown efficiency is consistent with previous findings by James et al., eLife [7], who reported a mild decrease in baseline EPSP amplitudes in *Plc21C* RNAi larvae compared to controls. In line with this observation, our electrophysiological recordings demonstrate that a comparable (~50-60%) reduction in *Plc21C* expression produces only subtle decreases in EPSP amplitudes and miniature event frequencies. Importantly, this modest reduction is sufficient to counterbalance the elevated mEPSP frequency and EPSP amplitudes observed in *Asap* mutants. Furthermore, GCaMP5G imaging revealed a similar pattern of phenotypic suppression upon *Plc21C* depletion in the *Asap* mutant background. These findings have been incorporated into the revised **Figure 5,** providing more substantial support for a functional interaction between Asap and Plc21C signaling.

We have also conducted comprehensive control experiments for *Arf6* RNAi and *Arf6^DN^* expression to evaluate NMJ morphology, active zone density, basal calcium levels, microtubule loop formation, and failure analysis. None of these parameters differed significantly from control NMJs. Notably, we observed only a mild increase in miniature frequency in both Arf6 RNAi and Arf6^DN^ larvae relative to Gal4 controls. These datasets are now included in **Figures 6 and 8, as well as in Supplemental Figure 8**. Additionally, short-term synaptic plasticity assays revealed no significant alterations in paired-pulse ratios at 0.1, 0.5, or 1.5 mM extracellular calcium across *Asap* mutants and rescue conditions. Given the absence of major phenotypic deviations, we did not extend these control experiments to **Supplemental Figure 5.**

To address the reviewer's concern regarding genetic background and driver effects, we systematically examined UAS-transgene and GAL4 driver lines independently in the *Asap* mutant background. Both genotypes exhibited NMJ morphology and electrophysiological profiles indistinguishable from those of *Asap* mutants, confirming that neither GAL4 nor UAS alone rescues the phenotype. These control data, now provided in **Supplemental Figure 3**, confirm that the observed effects are specific to Asap function and are not attributable to insertional artifacts or leaky transgene expression.

Overall, these additional experiments and controls further support the regulatory relationship between Asap-Arf6-PLCβ and substantiate our conclusion that Asap modulates synaptic transmission through IP3 signaling pathway.

**Minor point:**

***The authors attempted to perform epistatic analysis of Arf1 and dAsap in a dAsap mutant background by overexpressing Arf1-DN, but the animals did not survive to the 3rd instar larval stage. This situation can be circumvented by using the classical MARCM method. MARCM can generate dAsap mutant clones and simultaneously overexpress Arf1-DN and mCD8GFP in a Asap-/+ mosaic background. The desired clones can be identified by GFP+ expression.***

**Response:** We thank the reviewer for this insightful suggestion. We acknowledge that employing the MARCM technique could, in principle, enable the generation of *Asap* mutant clones that simultaneously overexpress *Arf1*-DN and mCD8-GFP in a *dAsap-/+* mosaic background, thereby facilitating an epistatic analysis at the single-cell level. However, due to the technical complexities associated with generating and maintaining multiple recombinant alleles and transgenes in postmitotic neurons, performing such experiments poses significant genetic and logistical challenges.

While we do not exclude a potential interaction between Arf1 and Asap, our current data support a more robust and direct functional association between Asap and Arf6, which appears to mediate synaptic stability and plasticity through a shared signaling pathway. Future studies utilizing advanced genetic tools or mosaic approaches may further elucidate whether Arf1 contributes to this regulatory network.

**Reviewer #4:**

The manuscript by Mallik et al addresses the role of Asap and Arf6 in modulating IP3-regulated calcium release at the larval NMJ. The authors generate *Asap* mutants and report morphological and electrophysiological phenotypes, including larger boutons with an increased number of active zones, increased mini frequency, and increased evoked responses. They correlate these defects with elevated cytoplasmic calcium levels at rest and confirm this by direct measurements with a GCaMP sensor. The authors use genetics, pharmacology, and electrophysiology to work through the relevant pathways downstream Asap. They report that Asap limits RyR, IP3, and PLCβ activities, which together control the release of calcium from the ER but not from mitochondria. Finally, the authors compare the Asap/IP3 signaling phenotypes with a combination of Asap/Arf6 l-o-f (Arf6 RNAi or a dominant negative transgene in the *Asap* mutant background). These epistasis experiments indicate that Asap l-o-f increases intracellular Ca2+ levels in an Arf6-dependent manner; the NMJ phenotypes observed in *Asap* mutants are also Arf6-dependent.

The findings that Asap/Arf6/IP3 signaling controls calcium release and thus regulates the NMJ function are novel and interesting. The genetics and epistasis arguments usually provide a powerful means to sort through various pathway components. But the authors omitted several experiments, so the reader is presented with correlational data rather than direct epistasis results. For example, the direct connection between Arf6 and IP3 is inferred but never tested directly. There are also a number of incorrect statements and panels that must be revised before this manuscript can be considered for publication.

**Major issues:**

***1) The epistasis experiments are incomplete. The authors showed two panels with Arf6-CA overexpression in Asap mutants (Figure 6C), but they do not mention these results in the manuscript. This Arf6-CA transgene must be overexpressed by itself, in a wild-type background, and the NMJ***

***morphology and function of these animals (i.e., D42> Arf6-CA) must be compared to that of Asap l-o-f***

**Response:** We thank the reviewer for this insightful comment. We have now clarified the results of the Arf6^CA^ overexpression experiments in the main text. Expression of Arf6^CA^ in the *Asap* mutant background did not restore NMJ morphological defects to control levels, consistent with the idea that the loss of the ArfGAP domain in *Asap* elevates Arf6-GTP activity at mutant NMJs. Based on this hypothesis, we examined whether expression of constitutively active Arf6^CA^ in a wild-type background phenocopies *Asap* loss-of-function. Indeed, Arf6^CA^ overexpression led to increased miniature frequency, elevated EPSP amplitude, increased active zone density, and NMJ morphological abnormalities similar to those observed in *Asap* mutants. These findings support the notion that excessive Arf6 activation underlies the *Asap* mutant phenotype. The corresponding data are now included in **Figures 6 and 9.**

***2) The Arf6-CA transgene should also be combined with IP3-sponge, Ryr-RNAi, and plc-beta RNAi to directly test the epistatic relationships between Arf6 and the other pathway components. As it stands now, the authors show no direct connection between Arf6 and IP3, etc. Epistasis must be included here to support the proposed model.***

**Response:** We thank the reviewer for this insightful suggestion. In response, we have conducted epistatic analyses by co-expressing Arf6^CA^ with *UAS-IP3-sponge*, *itpr*-RNAi, *RyR*-RNAi, and *Plc21C*-RNAi to directly examine the functional interactions between Arf6 and key components of the ER-PLCβ signaling pathway. Quantitative assessments of miniature frequency, EPSP amplitude, and NMJ morphology revealed that the elevated synaptic activity and structural abnormalities induced by Arf6^CA^ overexpression were significantly suppressed upon co-expression with these genetic modifiers. These results indicate that the Arf6-CA phenotype depends on IP3R, RyR, and PLCβ-mediated signaling and phenocopies the *dAsap* mutant phenotype. The corresponding data are presented in new **Figure 9**. These new findings provide direct evidence supporting the proposed Asap-Arf6-PLCβ signaling pathway in regulating synaptic transmission.

***3) The authors don't provide any explanation for the increased Brp puncta. This is unfortunate and should be addressed.***

***For example, a paper that the authors cite, Pelletan et al, 2015, showed that active ARF6 increases the exchange of GDP for GTP on Rab3A. In addition, it was previously demonstrated that Rab3 (the fly ortholog of vertebrate Rab3A) dynamically controls the composition of the presynaptic release machinery at larval NMJ, including the Brp levels (PMID:20005823). This may explain why Asap l-o-f (and therefore Arf6 g-o-f) mutants have an increased number of Brp puncta. A somewhat satisfying experiment would be to monitor Rab3 levels across all genetic manipulations aimed at examining Brp puncta. The published antibody is available upon request.***

**Response:** We thank the reviewer for this insightful comment and for highlighting potential mechanisms underlying the increased Brp puncta observed in *Asap* mutants. The elevation in Brp puncta number in *Asap* mutants and Arf6^CA^ overexpression animals may result from multiple factors: (1) increased intracellular Ca²⁺ concentrations, as previously reported [4] and (2) altered regulation of Rab3, which dynamically controls the molecular composition of the active zone, including Brp levels [5]. As suggested by the reviewer, we examined Rab3 expression in both *Asap* mutants and Arf6^CA^ overexpression animals. While we observed reduced Rab3 levels in *Asap* mutants, Arf6^CA^ overexpression increased Rab3 levels, despite both conditions showing elevated active zone density. These results suggest that increased Brp puncta in *Asap* mutants are potentially linked to altered intracellular Ca²⁺ dynamics. The 50% Rab11, combined with elevated calcium, is sufficient to increase the number of Brp punctae, whereas Arf6-CA

may regulate active zone organization through Rab3-dependent pathways, consistent with the findings of Pelletan et al. [8]. The corresponding data are provided in **Supplemental Figures 8 and 9.**

***4) On the same topic, ideally, the authors should include a simple set of experiments confirming (or infirming) a direct connection between Arf6 and Rab3 in causing the Brp phenotypes reported here. These results will tremendously strengthen the author's arguments and also take care of a line of observations left unexplained.***

**Response:** We thank the reviewer for this valuable suggestion. To address this point, we have conducted epistatic interaction experiments combining *Rab3* mutants with Arf6^CA^ and Arf6 RNAi. These new experiments reveal that both *Arf6CA* and *Arf6* RNAi expression in the *Rab3* mutant background produce active zone phenotypes comparable to those observed in *Rab3* mutants alone. These results indicate that *Rab3* likely functions downstream of *Arf6*, consistent with the findings of Pelletan et al., who demonstrated that active Arf6 promotes GDP-to-GTP exchange on Rab3A. Collectively, our data support a functional link between Arf6 and Rab3A in regulating active zone density at larval NMJs. These results have been incorporated into the **new Figure 7.**

***5) Fig. 3C-J/ Table S3***

***The statement "Presynaptic homeostatic plasticity remains intact in Asap mutant" is incorrect and should be revised. Based on the values provided in Table S3, the Asap l-o-f larvae have a 1.34-fold increase in QC in response to PhTx. This is less than the required 1.5-fold, which is the classic threshold used to mark the expression of PHP. Thus, the Asap mutants are in fact PHP-deficient. This fact provides an even stronger argument for seeking alternative explanations for the observed increase in basal neurotransmission. The authors must correct this paragraph. Please report QC ratios for clearer results.***

**Response:** We thank the reviewer for this insightful comment. According to Frank et al. Neuron, 2006 [9], presynaptic homeostatic potentiation (PHP) is defined as "a compensatory increase in presynaptic neurotransmitter release, measured as an elevation in quantal content, that occurs following a reduction in postsynaptic receptor function to stabilize synaptic strength." In our study, PhTx application in *Asap* mutant larvae resulted in a significant 33% (1.34-fold) increase in quantal content relative to untreated *Asap* mutants. Although this increase does not reach the arbitrary 1.5-fold level referenced by the reviewer, this numerical ratio has not been established as a universal threshold for defining PHP expression in the field. Rather, PHP is recognized by a statistically significant compensatory increase in presynaptic release following postsynaptic perturbation. Therefore, our data indicate that *Asap* mutants retain a measurable and significant capacity for homeostatic potentiation and are not PHP-deficient. We have now included the quantal content ratios in the revised text and **Table S3** for clarity.

***Other concerns:***

***1) Fig. 1A- please show the gene diagram to scale; include a scale bar.***

***Also, please add S2A as an additional panel in the main Fig. 1. This will facilitate readers' understanding of the narrative related to Asap domains and the antigen used for antibody generation.***

**Response:** We thank the reviewer for the helpful suggestion. A scale bar has now been added to the gene diagram in Figure 1A to indicate relative genomic distances. In addition, the domain organization of the Asap protein, previously shown in Figure S2A, has been incorporated as an additional panel in the main Figure 1 (**Figure 1B**) to improve clarity and provide a more cohesive understanding of the Asap domains and the antigen region used for antibody generation.

***2) The Brp puncta appear similar between Asap mutants and wt control in Fig. 2I, but they are much more intense in the Asap mutants in Fig. 3M. This is concerning.***

***Also, in both Figs 2I and 3M, the authors must report the intensity of Brp puncta not only the puncta number per NMJ. Please change panel 2I with one from a specimen that is not stretched. As is, this panel is confusing. Please add an additional Brp-Fire-LUT panel in Fig 2H-J; this will capture the Brp intensities.***

**Response:** We thank the reviewer for these valuable comments. We have now quantified Brp fluorescence intensities in both control and *Asap* mutant NMJs. Quantitative analysis revealed a ~45% reduction in Brp intensity in *Asap* mutants compared to controls, indicating decreased active zone protein levels. To improve data visualization, we have incorporated an additional Brp-Fire-LUT panel in **Figure 2**, which more clearly depicts Brp intensity distributions.

Regarding Figure 2I, no significant difference in Brp intensity was observed between genotypes in this dataset, as the images were acquired under different laser settings and intended primarily to illustrate active zone density rather than intensity.

In Figure 3, the experiments were designed using two independent sample sets: one containing unchallenged and PhTx-challenged control fillets, and the other containing unchallenged and challenged *Asap* mutant fillets. Comparisons were made within each genotype, not across them. Consistent with established homeostatic plasticity responses, Brp intensity increased significantly in the challenged condition compared with the unchallenged condition in both genotypes. Therefore, a direct comparison between the unchallenged control and the unchallenged *Asap* mutant fillets in Figure 3 is not appropriate. We have now clarified this in the materials and methods section.

Finally, the NMJ image shown in Figure 2I is not physically stretched; similar morphological features were consistently observed across multiple mutant samples. The apparent "stretching" likely reflects an expansion of the postsynaptic glutamate receptor fields, which may alter the perceived organization of the presynaptic Brp puncta.

***3) In Fig. 2M, the authors plot GluRIII cluster area, even though they probably measured the cluster diameter. (The Materials and Methods lack any description for these metrics.) Please report the GluRIII cluster diameter - it doesn't help to exacerbate the difference.***

**Response:** We thank the reviewer for this comment. The GluRIII clusters in the larval NMJ were quantified by generating a binary mask in ImageJ to delineate the boundaries of each punctum apposed to active zone markers. From this mask, the cluster area (µm²) of each cluster was measured. In response to the reviewer's suggestion, the methods section has been updated to clearly describe the metrics and the image analysis workflow.

***4) Show muscle resistance for all the electrophysiology recordings in the supplementary tables. Also, indicate the extracellular Ca2+ concentrations.***

**Response:** As stated in the materials and methods section, only samples with muscle input resistance greater than 5 MΩ were included in the electrophysiological quantification. The following have been mentioned in the supplemental table legends. We have now provided the complete dataset, including calcium concentrations in the corresponding supplemental electrophysiology tables.

***5) The synapse diagrams are colored in blue/white (Fig. 3), blue/blue (Fig. 4), white (Fig. 5A) or pink/pink (Fig. 8). This is neither appropriate nor useful. Please keep things consistent. Also, many of these diagrams do not need the muscle side. Please simplify.***

**Response:** We have now simplified the diagram and used blue consistently across all figures.

***6) Fig. 3A and the associated main text introduce GluRIIA as the only receptor affected by PhTx. This is incorrect (please see PMID: 39602131). Please revise and also simplify the diagram accordingly.***

**Response:** We thank the reviewer for pointing this out. The original study by Frank et al. [9] demonstrated that mEPSP amplitudes and frequencies in *GluRIIASP16* homozygous mutants were not further reduced upon PhTx treatment, suggesting that PhTx predominantly affects GluRIIA-containing receptors at the *Drosophila* NMJ. However, this does not exclude the potential contribution of GluRIIB-containing receptors. More recent work by Han TH et al. [10] indicates that PhTx can target both GluRIIA- and GluRIIB-containing receptors in heterologous HEK293T cells. Based on these findings, we have modulated our statement in the main text to reflect that PhTx affects GluR.

***7) Please revise the traces and data shown in Figure S4. They are not on the same scale.***

***The spacing between pulses (in panels C and E) varies. Also, the amplitudes of the traces shown in panel G do not match those plotted in panel H. For example, the last trace has an amplitude >40 mV, but there is no datapoint in panel H with such a large amplitude value for that genotype.***

**Response:** We have carefully reviewed the traces and revised them as suggested.

***8) The number of Brp puncta varies 2-fold between the Asap l-o-f larvae in Fig. 2K and the same genotype revisited in Fig. S5 panel J. This is concerning. Please explain.***

**Response:** We thank the reviewers for bringing this vital point to our attention. The experiments presented in Figures 2K and S5J (**now Supplemental Figure S8N**) were conducted independently under different rearing conditions. Specifically, larvae in Figure 2K were raised on standard cornmeal medium, whereas those in the latter experiment were raised on a molasses-enriched medium. Larvae reared on molasses-based food exhibited markedly improved health and overall robustness compared to those raised on cornmeal medium, which may partially account for the observed differences in Brp puncta. Nevertheless, in both experimental conditions, we consistently observed a statistically significant increase in Brp density in the mutant larvae compared to their respective controls.

***9) Please rephrase the sentence below for clarity.***

***"The multidomain containing ArfGAP with SH3 domain, Ankyrin repeat and BAR domain Protein (ASAP) subfamily proteins, which contain a BAR and an ArfGAP domain, hydrolyze Arf6.GTP through its GTPase-activating activity in vitro (Ismail et al., 2010; Kushwaha et al., 2024)."***

**Response:** Now we have rewritten the statement as "The ASAP subfamily proteins are multidomain ArfGAPs that contain SH3, Ankyrin repeat, BAR, and ArfGAP domains. They catalyze the hydrolysis of GTP bound to Arf6 through their intrinsic GTPase-activating activity *in vitro*. The above statement has been updated in the main text.

***10) To troubleshoot the anti-Asap high background staining, the authors should try to pre-adsorb their sera with Asap l-o-f larvae.***

**Response:** We thank the reviewers for their suggestion and performed similar experiments as suggested, but we still observed high background staining. We generated peptide antibodies against the Asap protein (not shown in this manuscript), but these antibodies also could not eliminate the background.

***11) pg. 12- Remove "from ER" from the sentence below, as this was not addressed yet up to this point. "Together, these results are consistent with the idea that elevated intracellular calcium release from ER and increased active zone numbers in Asap mutants contribute to maintaining robust neurotransmission."***

**Response:** We thank the reviewer for this comment. Based on additional epistatic genetic experiments, we now provide evidence that ER-mediated calcium release through itpr/RyR contributes to the elevated intracellular calcium observed in *Asap* mutants.

**References**

1.	Zengel JE, Sosa MA, Poage RE, Mosier DR. Role of intracellular Ca2+ in stimulation-induced increases in transmitter release at the frog neuromuscular junction. J Gen Physiol. 1994;104(2):337-55. doi: 10.1085/jgp.104.2.337. PubMed PMID: 7807052; PubMed Central PMCID: PMCPMC2229208.

2.	Goswami SP, Bucurenciu I, Jonas P. Miniature IPSCs in hippocampal granule cells are triggered by voltage-gated Ca2+ channels via microdomain coupling. J Neurosci. 2012;32(41):14294-304. doi: 10.1523/JNEUROSCI.6104-11.2012. PubMed PMID: 23055500; PubMed Central PMCID: PMCPMC3632771.

3.	Brusich DJ, Spring AM, James TD, Yeates CJ, Helms TH, Frank CA. Drosophila CaV2 channels harboring human migraine mutations cause synapse hyperexcitability that can be suppressed by inhibition of a Ca2+ store release pathway. PLoS Genet. 2018;14(8):e1007577. Epub 20180806. doi: 10.1371/journal.pgen.1007577. PubMed PMID: 30080864; PubMed Central PMCID: PMCPMC6095605.

4.	Mallik B, Bhat SA, Wang X, Frank CA. Mitochondrial Complex I and ROS control neuromuscular function through opposing pre- and postsynaptic mechanisms. PLoS Biol. 2025;23(9):e3003388. Epub 20250922. doi: 10.1371/journal.pbio.3003388. PubMed PMID: 40982549; PubMed Central PMCID: PMCPMC12478897.

5.	Graf ER, Daniels RW, Burgess RW, Schwarz TL, DiAntonio A. Rab3 dynamically controls protein composition at active zones. Neuron. 2009;64(5):663-77. doi: 10.1016/j.neuron.2009.11.002. PubMed PMID: 20005823; PubMed Central PMCID: PMCPMC2796257.

6.	Vajente N, Norante R, Redolfi N, Daga A, Pizzo P, Pendin D. Microtubules Stabilization by Mutant Spastin Affects ER Morphology and Ca(2+) Handling. Front Physiol. 2019;10:1544. Epub 20191220. doi: 10.3389/fphys.2019.01544. PubMed PMID: 31920731; PubMed Central PMCID: PMCPMC6933510.

7.	James TD, Zwiefelhofer DJ, Frank CA. Maintenance of homeostatic plasticity at the Drosophila neuromuscular synapse requires continuous IP(3)-directed signaling. Elife. 2019;8. Epub 20190610. doi: 10.7554/eLife.39643. PubMed PMID: 31180325; PubMed Central PMCID: PMCPMC6557630.

8.	Pelletan LE, Suhaiman L, Vaquer CC, Bustos MA, De Blas GA, Vitale N, et al. ADP ribosylation factor 6 (ARF6) promotes acrosomal exocytosis by modulating lipid turnover and Rab3A activation. J Biol Chem. 2015;290(15):9823-41. Epub 20150220. doi: 10.1074/jbc.M114.629006. PubMed PMID: 25713146; PubMed Central PMCID: PMCPMC4392280.

9.	Frank CA, Kennedy MJ, Goold CP, Marek KW, Davis GW. Mechanisms underlying the rapid induction and sustained expression of synaptic homeostasis. Neuron. 2006;52(4):663-77. doi: 10.1016/j.neuron.2006.09.029. PubMed PMID: 17114050; PubMed Central PMCID: PMCPMC2673733.

10.	Han TH, Vicidomini R, Ramos CI, Mayer ML, Serpe M. The gating properties of Drosophila NMJ glutamate receptors and their dependence on Neto. J Physiol. 2024;602(24):7043-64. Epub 20241127. doi: 10.1113/JP287331. PubMed PMID: 39602131; PubMed Central PMCID: PMCPMC11649521.
