# Supplementary material for: An ArfGAP-dependent signaling modulates synaptic plasticity via IP3-regulated calcium release from the endoplasmic reticulum
Source: PLoS Genet. 2026 Jan 23;22(1):e1012031. doi: 10.1371/journal.pgen.1012031 (PMC12863683; doi:10.1371/journal.pgen.1012031)
Supplement: Attachment — Submitted filename: Response_PLOS Genetics R2_03012026.docx [file pgen.1012031.s016.docx]

Please find below our detailed point-by-point responses to other reviewer's comments:

**Manuscript ID: PGENETICS-D-25-00364**

We thank all reviewers for their careful evaluation of our revised manuscript. Their constructive feedback has significantly strengthened the quality and clarity of this study. In response to the remaining minor concerns raised by reviewers 1 and 4, we have made the following changes and revised manuscript.

**Reviewer #1:**

**Minor Point**

1. *Overall, the revised manuscript has been improved, and the authors have addressed the majority of my comments. I do think the manuscript would have been further strengthened by looking at presynaptic calcium levels in response to stimulation to determine if changes in presynaptic calcium were responsible for the increased EJP amplitudes.*

**Response:** We thank the reviewer for this critical comment regarding the link between presynaptic calcium dynamics and the increased evoked neurotransmission observed in *Asap* mutants. While we initially stated that elevated basal presynaptic calcium levels in *Asap* mutants increase mEPSP frequency, subsequent genetic and pharmacological manipulations suggest that elevated basal calcium levels increase EPSP amplitudes and mEPSP frequencies. Now, in response to the reviewer’s suggestion, we have alternatively tested activity-induced calcium responses using the genetically encoded calcium indicator GCaMP5G. The ventral nerve cords were severed from the dorsal brain lobes to eliminate any inputs from the brain before the KCl stimulation. NMJ were stimulated with 90 mM KCl under low extracellular calcium (0.5mM). We observed a ~30% increase in GCaMP5G fluorescence in control boutons and a higher ~100% increase in *Asap* mutant boutons compared to their respective unstimulated larvae. These findings indicate that *Asap* loss significantly enhances presynaptic calcium response to stimulation, supporting the idea that the elevated EPSP amplitudes in *Asap* mutants likely arise due to altered calcium handling during synaptic activity. This data has now been incorporated as ***new Supplemental Figure S6*** in the revised manuscript. These new results strengthen the connection between elevated presynaptic calcium level and enhanced evoked neurotransmission in *Asap* mutants.

**Reviewer #4:**

**Minor Point**

1. *Congratulations on a solid piece of work. The revised manuscript is much improved.*

*However, the original EPSP trace in Fig 3E (but not the mEPSP) was replaced with a different trace, the amplitude of which is lower than any data point presented in the accompanying the bar graph (3I). Please correct.*

**Response:** We thank the reviewer for identifying this inconsistency. Now, we have replaced the EPSP trace in Figure 3E with a representative trace based on the mean amplitude of the data points shown in Figure 3I.
